# Kaposi's Sarcoma-Associated Herpesvirus Induces Nrf2 during *De Novo* Infection of Endothelial Cells to Create a Microenvironment Conducive to Infection

**DOI:** 10.1371/journal.ppat.1004460

**Published:** 2014-10-23

**Authors:** Olsi Gjyshi, Virginie Bottero, Mohanan Valliya Veettil, Sujoy Dutta, Vivek Vikram Singh, Leela Chikoti, Bala Chandran

**Affiliations:** H. M. Bligh Cancer Research Laboratories, Department of Microbiology and Immunology, Chicago Medical School, Rosalind Franklin University of Medicine and Science, North Chicago, Illinois, United States of America; University of Utah, United States of America

## Abstract

Kaposi's sarcoma-associated herpesvirus (KSHV) is the etiological agent of Kaposi's sarcoma (KS) and primary effusion B-cell lymphoma. KSHV induces reactive oxygen species (ROS) early during infection of human dermal microvascular endothelial (HMVEC-d) cells that are critical for virus entry. One of the downstream targets of ROS is nuclear factor E2-related factor 2 (Nrf2), a transcription factor with important anti-oxidative functions. Here, we show that KS skin lesions have high Nrf2 activity compared to healthy skin tissue. Within 30 minutes of *de novo* KSHV infection of HMVEC-d cells, we observed Nrf2 activation through ROS-mediated dissociation from its inhibitor Keap1, Ser-40 phosphorylation, and subsequent nuclear translocation. KSHV binding and consequent signaling through Src, PI3-K and PKC-ζ were also important for Nrf2 stability, phosphorylation and transcriptional activity. Although Nrf2 was dispensable for ROS homeostasis, it was essential for the induction of COX-2, VEGF-A, VEGF-D, Bcl-2, NQO1, GCS, HO1, TKT, TALDO and G6PD gene expression in KSHV-infected HMVEC-d cells. The COX-2 product PGE2 induced Nrf2 activity through paracrine and autocrine signaling, creating a feed-forward loop between COX-2 and Nrf2. vFLIP, a product of KSHV latent gene ORF71, induced Nrf2 and its target genes NQO1 and HO1. Activated Nrf2 colocalized with the KSHV genome as well as with the latency protein LANA-1. Nrf2 knockdown enhanced ORF73 expression while reducing ORF50 and other lytic gene expression without affecting KSHV entry or genome nuclear delivery. Collectively, these studies for the first time demonstrate that during *de novo* infection, KSHV induces Nrf2 through intricate mechanisms involving multiple signal molecules, which is important for its ability to manipulate host and viral genes, creating a microenvironment conducive to KSHV infection. Thus, Nrf2 is a potential attractive target to intervene in KSHV infection and the associated maladies.

## Author Summary

KSHV infection of endothelial cells *in vivo* causes Kaposi's sarcoma and understanding the steps involved in *de novo* KSHV infection of these cells and the consequences is important to develop therapies to counter KSHV pathogenesis. Infection of endothelial cells *in vitro* is preceded by the induction of a network of host signaling agents that are necessary for virus entry, gene expression and establishment of latency. Our previous studies have implicated reactive oxygen species (ROS) as part of this network. In the current study, we show that ROS activate Nrf2, a master transcriptional regulator of genes involved in ROS homeostasis, apoptosis, glucose metabolism and angiogenesis. Besides ROS, KSHV utilizes additional aspects of host signaling to induce Nrf2 activity. We also observed that infection of endothelial cells deficient in Nrf2 resulted in downregulation of multiple genes important in KSHV pathogenesis, such as COX-2 and VEGF, and affected proper expression of two hallmark KSHV genes, lytic ORF50 and latent ORF73. Taken together, this study is the first to demonstrate the importance of Nrf2 during *de novo* KSHV infection of endothelial cells, and establishes Nrf2 as an attractive therapeutic target to control KSHV infection, establishment of latency and the associated cancers.

## Introduction

Kaposi's sarcoma-associated herpesvirus (KSHV) or human herpesvirus 8 (HHV-8), a γ-2 lymphotropic herpesvirus with a double-stranded DNA genome of ∼160 kb in length, is the etiological agent of hyper-proliferative disorders such as Kaposi's sarcoma (KS), primary effusion B-cell lymphoma (PEL), and plasmablastic multicentric Castleman's disease (MCD) [1]–[3]. KS lesions exhibit a heterogeneous environment of hyperplastic, endothelium-derived spindle cells, neovascular structures and inflammatory cells [4]. Like all herpesviruses, the KSHV life-cycle alternates between lytic and latent phases, and KSHV is predominantly in the latent state in KS endothelial cells [5]. KSHV genome and transcripts are also detected in the KS lesion fibroblasts, monocytes, and cells of epithelial origin and the expression of multiple latent and lytic genes in the infected cells, aided by the concomitant action of pro-inflammatory cytokines released by these cells, drives the excessive proliferation and hyperplasia of endothelial cells that lead to their spindle-shaped morphology [5].

Investigation of KSHV infection of endothelial cells is frequently carried out *in vitro* in primary endothelial cell types such as human dermal microvascular endothelial cells (HMVEC-d), human umbilical vein endothelial cells (HUVEC) and lymphatic endothelial cells (LEC), or in immortalized endothelial cell-lines such as TIVE/TIVE-LTC and epithelial SLK/iSLK cells. HMVEC-d cells provide an excellent *in vitro* model for studying the early events that follow *de novo* infection of endothelial cells because i) they are naïve, primary cells permissive to KSHV infection, ii) they are derived from the same cells that eventually transform into the characteristic spindle-shaped morphology in KS lesions, and iii) are not transformed, hence, exhibit signaling cascades that closely resemble early events during *in vivo* infection [6]. As primary cells, HMVEC-d cells have a limited life-span and culturing them is labor intensive, with about ∼50–70% infection efficiency if ample virus is used, and exhibit progressive viral episome loss with each cellular division [5], [6].

The KSHV-binding receptor on HMVEC-d cells is heparan sulfate (HS), a negatively-charged plasma membrane macromolecule that uses electrostatic forces to attract KSHV envelope glycoproteins to the cell surface [7]–[11]. Once on the surface of the cells, KSHV envelope glycoproteins interact with entry receptors such as integrins (α3β1, αVβ3 and αVβ5), xCT/CD98, and the receptor tyrosine kinase EphA2 to induce important signaling pathways that result in the phosphorylation and activation of many additional kinases and transcription factors [8], [10]–[14]. Specifically, KSHV infection sequentially induces activation of FAK, Src, PI3-K/Akt, ROS, EphA2, c-Cbl and CIB1 to mediate macropinocytosis and virus entry [14]–[21]. Subsequently, infection induces activation of PKC-ζ, COX-2, MAPKs (MEK and ERK1/2) and NF-κB, which collectively create a microenvironment conducive to establishment of viral gene expression and latency [10], [22]–[25]. Unlike alpha- and beta-herpesviruses, whose *in vitro* infection results in robust lytic replication with high progeny virus formation and cytopathological changes in the cell, KSHV establishes latency within 24 hr post-infection (p.i.), which is clearly mirrored by a steady rise in major latency regulatory ORF73 (LANA-1) gene expression and with no progeny virus formation [5], [26]. Another interesting feature of *de novo* infection is that before the establishment of latency, a quick burst of lytic genes with important anti-apoptotic and immune-evasive roles peaks between 2–8 hr p.i., which subsequently declines by 24 hr p.i., likely due to i) increase in the antagonizing LANA-1 expression, ii) chromatin modifications of the KSHV genome, and iii) potentially other unidentified mechanisms [26].

We and others have also shown that stress-associated agents, such as reactive oxygen species (ROS), play important roles in KSHV pathogenesis. ROS have been shown to induce lytic reactivation of KSHV in latently-infected endothelial cells [27]–[29]. Our studies for the first time demonstrated that *de novo* KSHV infection of HMVEC-d cells induces ROS by ∼2-fold as early as 30 min p.i., which was sustained throughout the course of infection and during latency [16]. This induction played an important role in mediating phosphorylation of a signal pathway involved in macropinocytosis, virus entry and establishment of KSHV infection [16]. However, these studies did not examine the downstream effects of ROS activation.

Nuclear factor E2-related factor 2 (Nrf2) is a ROS-responsive, ∼65 kDa, master transcription factor involved in the transcriptional activation of hundreds of human genes [30]. Nrf2 belongs to the basic leucine zipper (bZIP) subset of the *cap ‘n’ collar* family of transcription factors and consists of six highly conserved Nrf2-ECH homology domains labeled Neh1-6 [30]. Neh1 contains the DNA-binding domain; Neh2 contains the ETGE and DLG motifs that bind to its inhibitor, Keap1 (Fig. S1); Neh3 is important for activity of the transactivation domains Neh4 and Neh5; Neh6 binds to GSK-3β and β-TrCP [31]–[40]. When activated, Nrf2 binds to DNA promoters that contain the anti-oxidant response element (ARE - TGAnnnnGC) and induces expression of genes such as NQO1, GCS, HO1, and GST etc. involved in the stress response [34], [41]–[45]. In addition, recent studies have determined that Nrf2 induces transcription of genes involved in drug clearance (Mrp1 and Mrp2) [46], [47], glucose and glutamine metabolism (G6PD, TKT, TALDO, PGD, ME1 etc.) [48], [49], apoptosis (Bcl-2, Bcl-xL) [50], [51], angiogenesis (HIF-1α and VEGF) [52]–[54] and cell invasion (MMP9) [55].

Nrf2 is the major regulator of ROS homeostasis in multiple cell types [56]. Because of the importance of responding to elevated ROS rapidly before irreversible cell damage ensues, the cells have evolutionarily developed a quickly-inducible system of Nrf2 activation. In a steady state, new Nrf2 molecules are constantly translated in abundance, but are quickly degraded by its inhibitor, kelch-like ECH-associated protein 1 (Keap1), which acts as a scaffold for the E3 ubiquitin ligase Cul3 (Fig. S1) [57]–[59]. Keap1 consists of three domains, i) the BTB domain (Broad Complex, Tramtack, and Bric-a-Brac), ii) the linker region heavy in reactive cysteine residues, and iii) the Kelch domain [60]. The N-terminal BTB domain homodimerizes with another Keap1 molecule and recruits Cul3 (Fig. S1A, i) [57]. In the closed conformation, each of the C-termini Kelch domains of a Keap1 homodimer binds to the ETGE or DLG domains of one Nrf2 molecule (Fig. S1A, ii) [61], [62]. The N-terminus-bound Cul3 then mediates ubiquitination of 7 lysine residues on the Neh2 domain of Nrf2 (Fig. S1A, ii), an event that opens the complex and allows 26S proteasomal degradation of Nrf2 and recycling of Keap1-Cul3 (Fig. S1A, iii) [58], [59], [63], [64]. Therefore, in a steady state, this system cycles between a closed conformation important for Nrf2 ubiquitination, and an open conformation important for Nrf2 degradation and Keap1-Cul3 recycling (Fig. S1A, overarching arrow) [61], [62]. The recycled Keap1-Cul3 complex then targets a newly synthesized Nrf2, restarting the cycle and keeping Nrf2 levels low.

When ROS levels are elevated, free radicals attack multiple cysteine residues on Keap1, leading to conformational and functional changes that disrupt the normal activity of the Keap1-Cul3 system (Fig. S1B) [60], [65]. Specifically, Nrf2 inducers “lock” the Keap1-Nrf2 interaction in the closed conformation, with both Kelch domains tightly bound to ETGE and DLG, irrespective of the ubiquitination status of Nrf2. As new Nrf2 is translated, the Keap1-Cul3 system is quickly saturated (Fig. S1B, ii and iii) and the newly synthesized Nrf2 accumulates in the cell, free of inhibition from the Keap1-Cul3 ubiquitination machinery (Fig. S1B, i) [61], [62]. The Nrf2 inducers tBHQ and sulforaphane have been shown to affect this pathway [59]. Once stabilized, Nrf2 is phosphorylated on its Ser-40 residue, which results in its nuclear translocation and transcriptional activation of multiple ARE-responsive genes [66]. Several kinases have been reported to either directly or indirectly affect Nrf2 activity, including ERK1/2, casein kinase 2 (CK-2), multiple PKCs and PI3-K/Akt (Fig. S1B, i) [67]–[72].

Several stimuli can induce Nrf2 activation, and virus infection is one of them. The Influenza A virus induced ROS, and as a consequence Nrf2 in alveolar epithelial cells, an event that mitigated the Influenza-induced alveolar toxicity [73]–[75]. Human cytomegalovirus (HCMV) also induced Nrf2 activity during infection of human foreskin fibroblasts, which enhanced the productivity of the infection by decreasing the cytopathic effects of the infection [76]. Hepatitis C virus (HCV), through its core, E1, E2, NS4B and NS5A proteins, induced Nrf2 activity via ROS-dependent and ROS-independent mechanisms involving multiple kinases [77]–[80]. Recently, the Marburg virus structural protein VP-24 has been shown to inhibit Keap1 activity and enhance Nrf2-mediated anti-inflammatory responses [81], [82].

Oxidative stress is essential in development of all four types of KS [83] and analysis of KS tissue sections in the present study detected enhanced Nrf2 activity. We hypothesized that KSHV infection of endothelial cells induces ROS levels to manipulate Nrf2. Studies conducted to test this hypothesis demonstrate for the first time that KSHV induces Nrf2 activity during *de novo* infection of HMVEC-d cells. KSHV binding, signaling, gene expression and cytokine induction play important roles in inducing Nrf2 activity by mediating its stability, Ser-40 phosphorylation and nuclear translocation. This induction required ROS upregulation as well the activity of a series of host kinases induced by KSHV. Nrf2 induction was essential for the transcriptional activation of multiple host and viral genes that play important functions in KSHV infection. Collectively, these data suggest that KSHV has developed multiple mechanisms to induce Nrf2 activity during *de novo* infection, establishing Nrf2 as an important agent in KSHV biology and KS pathogenesis.

## Results

### Kaposi's sarcoma lesions exhibit upregulated levels of Nrf2

**Figure 1:**
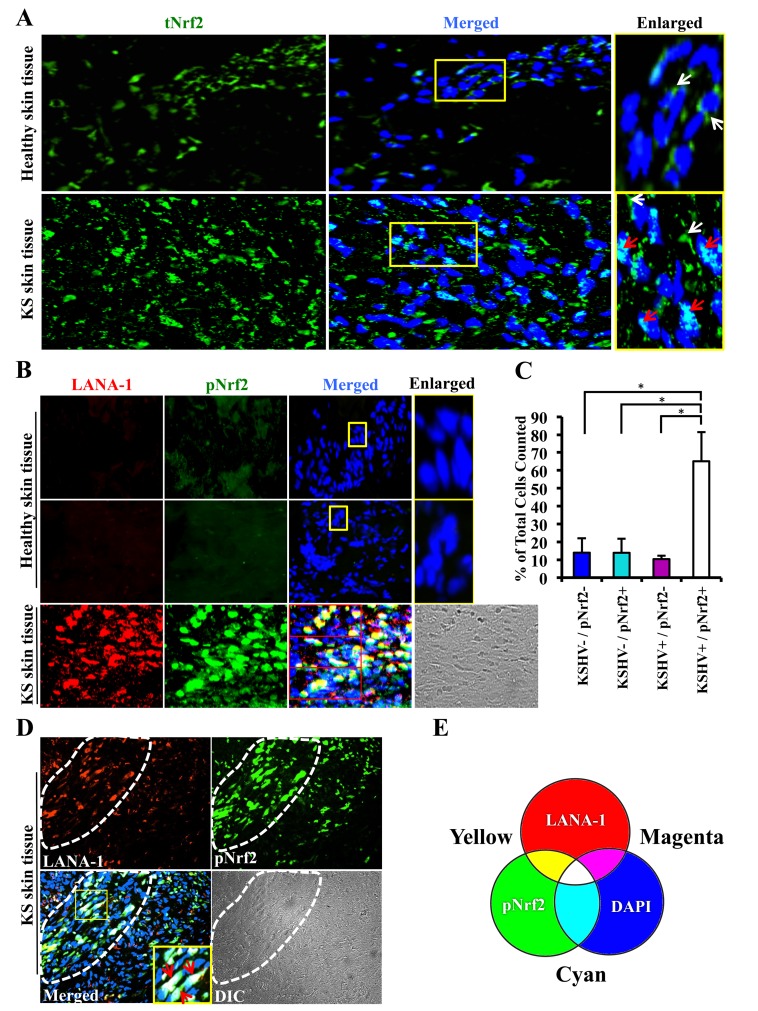
Immunofluorescence analysis of Nrf2 levels in Kaposi's sarcoma skin lesions. **A**) Healthy skin tissue (top row) and Kaposi's sarcoma skin tissue (bottom row) slides were assayed by immunofluorescence and incubated with rabbit anti-tNrf2 primary antibody and then with goat anti-rabbit (Alexa-Fluor 488-green) secondary antibody. DAPI was used to visualize the nuclei and the merged tNrf2/DAPI image is shown in the middle column. Yellow squares in the middle column indicate the area that has been enlarged in the right column. Red arrow = nucleus; white arrow = cytoplasm. **B**) Healthy skin (top two rows) and KS skin tissue (bottom row) were double-stained for LANA-1 (Alexa-Fluor 594- red) and host phosphorylated pNrf2 (Alexa-Fluor 488 – green). DAPI was used to visualize the nuclei, and the triple merge of LANA-1, pNrf2 and DAPI is shown in the third column. Yellow square = enlarged area. **C**) Quantitative representation of the colocalization of pNrf2, LANA-1 (KSHV+) and DAPI staining from the triple-merged figure of KS skin tissue in panel B. Cells that had detectable levels of staining of DAPI, pNrf2 and LANA-1 were considered as KSHV+/pNrf2+, while those staining only for DAPI were considered as KSHV−/pNrf2−. Bars indicate mean ± SD of 3 randomly selected fields containing at least 30 cells each (red boxes in Panel B). * = p<0.05. **D**) KS skin tissue containing an area dense in spindle cells (encircled by dashed line) and an area with low density of spindle cells (surrounding area). Yellow square indicates enlarged region; red arrow = triple colocalization. **E**) Venn diagram of the RGB color combination profiles.

Kaposi's sarcoma (KS) lesions consist of a heterogeneous environment of multiple cell types that include spindle-shaped endothelial cells, fibroblasts, monocytes, and neovascular structures [4]. At the molecular level, biochemical studies by us and others have demonstrated that KS lesions exhibit substantially elevated levels of pro-inflammatory and stress-associated agents like NF-κB, COX-2 and PGE2 [10], [22]–[25], [84], [85]. A hallmark of the stress response is the activation of the master regulator Nrf2 [56]. To determine whether KSHV-positive tissues exhibit enhanced Nrf2 activity, we performed an immunofluorescence assay (IFA). KS tissue samples exhibited substantially higher levels of Nrf2 compared to normal tissue (Fig. 1A). Moreover, while Nrf2 in healthy tissue localized mostly in the cytoplasm (Fig. 1A, top enlarged box, white arrows), we observed that Nrf2 localized predominantly in the nuclei of KS tissue cells (Fig. 1A, bottom enlarged box, red arrows) in addition to the cytoplasmic distribution (Fig. 1A, bottom enlarged box, white arrows).

Ser-40 phosphorylation of Nrf2 is an essential step in its activation that is required for its nuclear translocation and transcriptional activation, resulting in the augmentation of Nrf2-dependent gene expression [59]. To determine if the increased nuclear Nrf2 observed in KS tissues is of the phosphorylated, active form, we reacted the tissues with an antibody specific to Ser-40-phosphorylated Nrf2 (pNrf2). As expected, the level of pNrf2 in healthy tissue cells was undetectable in the nucleus or the cytoplasm (Fig. 1B, top two rows), indicating low Nrf2 activity. In contrast, KS tissue exhibited elevated levels of pNrf2, which predominantly colocalized with cells expressing latency-associated LANA-1, a common marker of KSHV infection (Fig. 1B, bottom row).

To determine if Nrf2 activation was linked with KSHV infection, we quantified the association between KSHV infection of a cell and its pNrf2 levels by counting all DAPI-staining cells in a particular field (red boxes on the bottom row of Fig. 1B) and considered that as the total number of cells within that field. Cells with only LANA-1 staining overlapping with DAPI were categorized as KSHV+/pNrf2−, while cells that displayed only detectable pNrf2 staining were categorized as KSHV−/pNrf2+. Cells with no staining other than DAPI were categorized as KSHV−/pNrf2−, while those that displayed a triple colocalization of the two proteins and DAPI were categorized as KSHV+/pNrf2+. By this method, >65% of the cells were positive for both LANA-1 and high levels of pNrf2 (Fig. 1C, KSHV+/pNrf2+). Fewer than 14% of the cells were LANA-1-deficient and exhibited high levels of pNrf2 expression (Fig. 1C, KSHV−/pNrf2+), while only 10% of the cells expressed LANA-1 but showed undetectable levels of pNrf2 (Fig. 1C, KSHV+/pNrf2−).

Because KS tissue may contain uninfected or non-endothelial bystander cells that may confound the observed increased Nrf2 activity, we compared the level of pNrf2 in KS areas abundant in spindle-shaped cells (Fig. 1D, area enclosed by dashed white line) to areas of tissue scarce in spindle-shaped cells (Fig. 1D, surrounding area). The spindle-shaped cells, in addition to their typical elongated nuclei, exhibited a high level of LANA-1 as well as pNrf2 staining in contrast to the cells in the surrounding areas (Fig. 1D). Moreover, mostly white staining, representing the triple-colocalization of pNrf2, LANA-1 and DAPI (Fig. 1E), was observed in the enlarged image of the merged panel (Fig. 1D, red arrows), which clearly suggested a predominantly nuclear localization of pNrf2 and LANA-1 in KS tissue.

Taken together, these results demonstrated that KS skin tissue exhibits elevated levels of active, nuclear Nrf2, and that this effect correlates strongly with KSHV infection.

### *De novo* KSHV infection of HMVEC-d cells induces Nrf2 levels and phosphorylation

**Figure 2:**
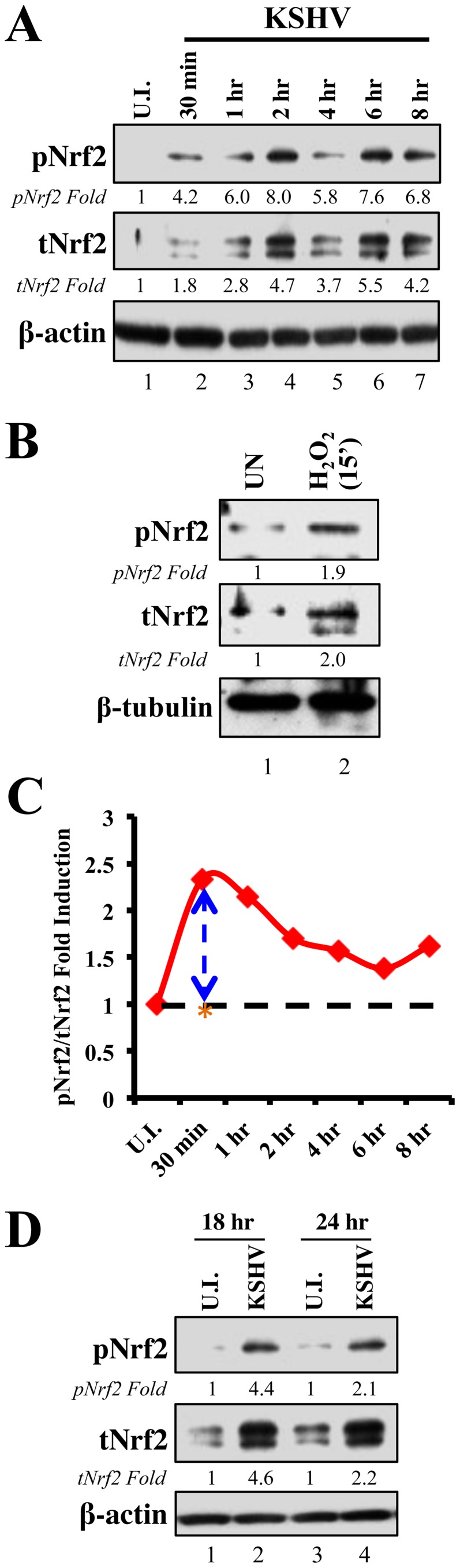
Induction of Nrf2 activity during *de novo* KSHV infection. **A**) Primary endothelial (HMVEC-d) cells starved for a total of 8 hr were infected with KSHV (20 DNA copies/cell) for the indicated time points and immunoblotted with pNrf2 and tNrf2 antibodies. β-actin was used as loading control. Fold inductions normalized to β-actin and relative to the uninfected (U.I.) condition (arbitrarily set to 1) are indicated. **B**) Starved HMVEC-d cells were treated with 100 µM H_2_O_2_ for 15 min prior to western blot analysis for pNrf2 and tNrf2. **C**) Graphical representation of the ratio of pNrf2/tNrf2 fold induction as observed in the Western blot illustrated in figure 1A. Dashed line = hypothetical line depicting constitutive phosphorylation; red line = actual ratio of pNrf2/tNrf2 during KSHV infection; orange asterisk = ratio of pNrf2/tNrf2 from H_2_O_2_ treatment. **D**) Starved HMVEC-d cells were infected with KSHV for 2 hr, incubated for 16 hr in growth factor-supplied media prior to additional starvation for 8 hr, at which point they were immunoblotted for pNrf2 and tNrf2.

Since KSHV-infected tissues had increased Nrf2 activity, we next determined whether *de novo* KSHV infection induces Nrf2 activity in endothelial cells. KSHV infection of HMVEC-d cells induces a signaling cascade that promotes virus entry, nuclear delivery and viral gene expression [8], [10]–[14]. To determine and quantitate virus binding and entry-mediated signaling pathways, we serum-starved HMVEC-d cells for 8 hr to reduce the signals induced by the serum (growth factors) in the culture medium. We then infected the cells for the indicated time points, and the levels of total Nrf2 (tNrf2) and pNrf2 were assessed by immunoblot analysis. We observed a 1.8-fold induction of tNrf2 as early as 30 min p.i., which steadily increased to ∼5-fold at 2 hr p.i. and was sustained during the monitored period of 8 hr p.i. (Fig. 2A, second panel). We also observed a robust ∼4-fold induction of pNrf2 as early as 30 min p.i., which peaked to 8-fold at 2 hr p.i. and was sustained during 8 hr p.i. (Fig. 2A, first panel). PKC-ζ phosphorylation was used as an infection marker, as it has been previously shown to be induced during *de novo* KSHV infection (Fig. S2A) [24]. To determine whether ROS can activate Nrf2 in HMVEC-d cells, we treated the cells with H_2_O_2_, a member of the ROS family, and observed an induction in tNrf2 levels to a similar magnitude, indicating a possible role for ROS in tNrf2 accumulation during KSHV infection (Fig. 2B, middle panel). H_2_O_2_ also induced pNrf2 by a similar magnitude as tNrf2 (∼1.9 vs. 2.0-fold at 15 min) (Fig. 2B), suggesting that HMVEC-d cells have a constitutive pathway that readily phosphorylates Nrf2 into pNrf2.

To determine whether the increase in pNrf2 observed during KSHV infection is an indirect result of constitutive tNrf2 phosphorylation, or due to an increase in activity of KSHV-induced kinases during early infection, the ratio of fold induction of pNrf2 to tNrf2 was plotted for each time point (Fig. 2C). If the induction of Nrf2 phosphorylation was constitutive, as with H_2_O_2_, one would expect the ratio to be ∼1.0 (Fig. 2C, dashed horizontal line). However, the actual ratio of pNrf2/tNrf2 fold induction was significantly higher than 1.0 throughout the observed course of early infection, 2.3-fold higher at 30 min p.i., leveling to ∼1.6-fold higher at 2 hr p.i. and thereafter (Fig. 2C, red line). These results suggested that Nrf2 phosphorylation during KSHV infection involves signaling kinase(s) induced by *de novo* KSHV infection. The kinetics of Nrf2 phosphorylation are consistent with KSHV signaling kinetics, where signaling occurs early during KSHV entry, followed by a series of cytokine releases that mediate a second wave of signaling [23]. The biology of *de novo* KSHV infection differs widely between early infection, where events are driven by virus-host surface receptor interactions, and late infection, where events are driven by the expression of a few KSHV latent genes. To assess whether latent KSHV infection also induces Nrf2 activation, we performed a Western blot analysis on HMVEC-d cells infected for 18 and 24 hr. We observed a 4.6 and 2.2-fold induction of tNrf2 and a 4.4 and 2.1-fold induction of pNrf2 at 18 and 24 hr p.i., respectively (Fig. 2D).

Collectively, these results demonstrated that *de novo* KSHV infection of HMVEC-d cells induced tNrf2 accumulation and its Ser-40 phosphorylation during the early and late stages of infection.

### *De novo* KSHV infection of HMVEC-d cells increases nuclear pNrf2

**Figure 3:**
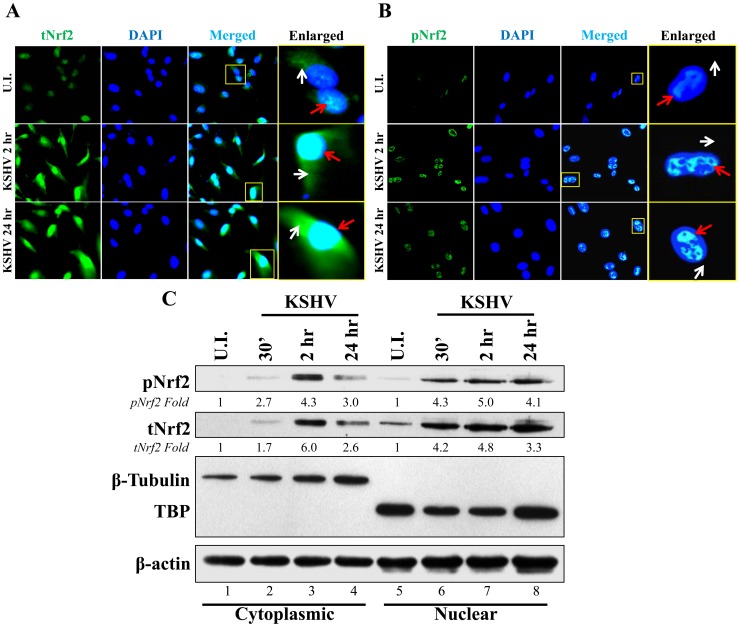
Nuclear localization of Nrf2 during KSHV infection. **A** and **B**) Nrf2 localization and levels during KSHV infection (20 DNA copies/cell) were visualized by IFA. Starved cells were infected with KSHV for 2 and 24 hr and stained for tNrf2 (**A**) or pNrf2 (**B**). DAPI was used to visualize the nuclei and merged tNrf2 or pNrf2/DAPI are shown in the third columns of the respective figure. Yellow square = enlarged area; red arrow = nuclear localization; white arrow = cytoplasmic localization. **C**) HMVEC-d cells were starved and infected as previously described, and the cytoplasmic and nuclear proteins were fractionated and then immunoblotted for pNrf2 and tNrf2. β-tubulin was used as a cytoplasmic purity control while TATA Binding protein (TBP) was used as a nuclear purity control. Fold inductions normalized to β-actin and relative to the uninfected (U.I.) condition (arbitrarily set to 1) are indicated.

Phosphorylation of Nrf2 is an important step that leads to its nuclear localization and increased transcriptional activity [59]. IFA of uninfected cells and cells infected with KSHV for 2 and 24 hr showed that tNrf2 was elevated throughout the cell (cytoplasm and nucleus) during the infection (Fig. 3A, red and white arrows). pNrf2, on the other hand, accumulated predominantly in the nuclei of infected cells both at 2 and at 24 hr p.i. (Fig. 3B, red arrows), while no cytoplasmic accumulation was observed (Fig. 3B, white arrows). Western blotting of fractionated cytoplasmic and nuclear protein from uninfected and infected cells corroborated the IFA data. Specifically, the levels of tNrf2 were induced by 1.7, 6.0 and 2.6-fold in the cytoplasm at 0.5, 2 and 24 hr p.i., respectively, while pNrf2 was induced by 2.7, 4.3 and 3.0-fold at the same time points (Fig. 3C, lanes 1–4). The nuclear protein fraction also exhibited a robust increase in tNrf2 by 4.2, 4.8 and 3.3-fold and pNrf2 by 4.3, 5.0 and 4.1-fold at 0.5, 2 and 24 hr p.i., respectively (Fig. 3C, lanes 5–8). Interestingly, although IFA at 2 hr p.i. showed predominantly nuclear pNrf2, the western blot assay demonstrated both nuclear and cytoplasmic pNrf2 accumulation at the same time point. The explanation for this discrepancy remains elusive, and is likely due to the different sensitivity of the pNrf2 antibody used in these different techniques. Taken together, these results demonstrated that Nrf2 induction during *de novo* KSHV infection of HMVEC-d cells leads to nuclear accumulation of pNrf2.

### *De novo* KSHV infection does not induce a high level of Nrf2 transcription

Because of the necessity to respond to stress in a rapid manner, cells constitutively translate new Nrf2 protein. However, under unstressed conditions, these cells maintain low Nrf2 protein levels by shunting it towards the proteasome through the ROS-dependent Keap1-Cul3 ubiquitination axis [59]. In this complex, Keap1 acts as a scaffolding protein for the Cul3 E3 ubiquitin ligase, which mediates lysine-48 ubiquitination of Nrf2 [57]–[59]. Once a targeted Nrf2 has been amply ubiquitinated, this complex releases it for proteasomal degradation, and scavenges for another newly synthesized Nrf2 [33], [58], [63], [64]. When ROS levels are elevated, conformational changes in Keap1 disrupt the axis, Cul3 cannot ubiquitinate newly synthesized Nrf2, which quickly accumulates inside the cell instead of being degraded by the proteasome [60]–[62], [65]. This leads to an appropriate anti-oxidative response achieved through a prompt Nrf2 increase and transcriptional upregulation of its target genes.

**Figure 4:**
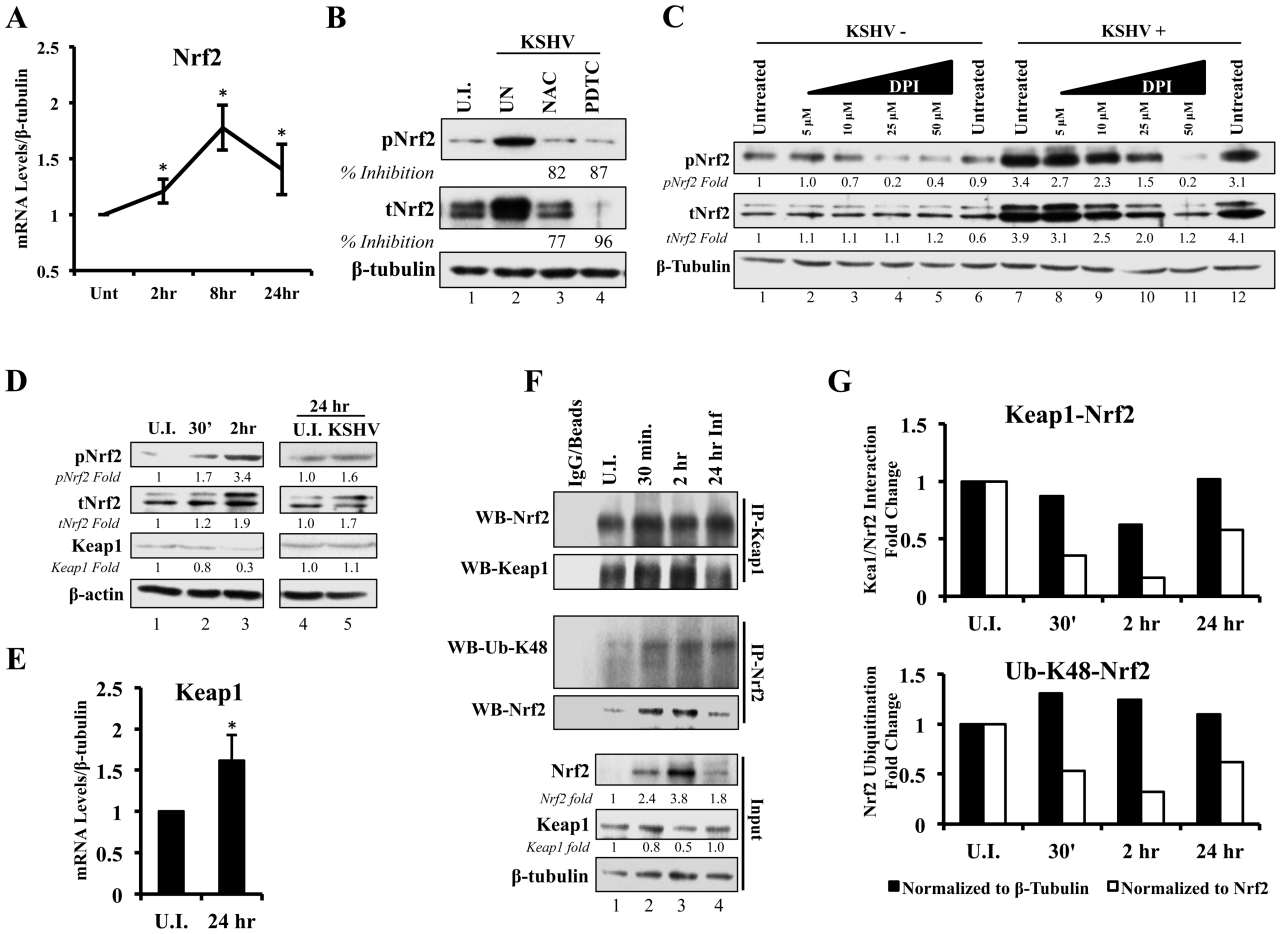
Effect of KSHV ROS induction on Nrf2-Keap1 interaction and Nrf2 ubiquitination. **A**) Real-time RT-PCR analysis of Nrf2 mRNA measured at various times post-infection of HMVEC-d cells. β-tubulin was used as an endogenous house-keeping gene. Each point represents fold induction compared to uninfected cells (Unt; arbitrarily set to 1) ± SD for 4 independent experiments. * = p<0.05. **B**) Starved HMVEC-d cells were treated with NAC (10 mM) or PDTC (100 µM) for 2 hr prior to infection with KSHV (20 DNA copies/cell) for an additional 2 hr before immunoblot analysis for tNrf2 and pNrf2. NAC = N-Acetylcysteine; PDTC = pyrrolidine dithiocarbamate. **C**) Starved HMVEC-d cells were treated with increasing concentration of DPI (0–50 µM) for 2 hr prior to infection with KSHV (20 DNA copies/cell) for an additional 2 hr before immunoblot analysis. DPI – Diphenyleneiodonium. **D**) Infected HMVEC-d cells were immunoblotted for pNrf2, tNrf2 and Keap1. Fold inductions normalized to β-actin and relative to the uninfected (U.I.) condition (arbitrarily set to 1) are indicated. **E**) Real-time RT-PCR analysis of Keap1 mRNA measured 24 hr p.i. from HMVEC-d cells infected with KSHV (20 DNA copies/cell). β-tubulin was used as endogenous house-keeping control. Bars represent fold change ± SD for 3 independent experiments. * = p<0.05. **F**) HMVEC-d cells were infected for the indicated time points and lysed with NETM buffer to isolate whole cell protein. Equal amounts of protein (200 µg/condition) were immunoprecipitated using anti-Keap1 or anti-Nrf2 antibody O/N at 4°C and immunoblotted for tNrf2 and Keap1 or Lysine-48-linked Ubiquitin (Ub-K48) and tNrf2, respectively. Bottom three panels indicate the whole cell lysate levels of Nrf2, Keap1 and β-tubulin. **G**) The pull-down results were normalized to either whole cell lysate (input) β-tubulin (black bars) or Nrf2 (white bars), and fold inductions relative to the uninfected conditions (U.I. - arbitrarily set to 1) are indicated. The Keap1-Nrf2 interaction is shown in the top graph, and the Nrf2 ubiquitination levels are shown in the bottom graph.

A KSHV-mediated increase in Nrf2 protein levels, as shown in figures 2 and 3, could be either due to upregulation of Nrf2 transcription, or due to modulation of Nrf2 protein stability through destabilization of the Keap1-Cul3 axis. To determine whether KSHV induces total Nrf2 levels by increasing its transcriptional levels, we performed real-time RT-PCR analysis using Nrf2-specific primers. We observed a 1.2, 1.8 and 1.4-fold induction of Nrf2 mRNA at 2, 8 and 24 hr p.i. (Fig. 4A). While statistically significant, the fold induction of the transcript is substantially lower than the fold induction of Nrf2 protein observed in figure 2A, explaining only in part the observed increase in total Nrf2 protein. Moreover, because the induction of Nrf2 protein preceded the induction of its transcript, the increase in Nrf2 transcript is likely a consequence and not a significant cause of increased Nrf2 protein. This is consistent with previous studies that have established the Nrf2 gene promoter as a target of Nrf2 transcriptional activity [86].

### ROS induction during *de novo* infection is essential for Nrf2 upregulation

Since transcriptional upregulation may not be the only reason for the observed Nrf2 protein increase, we focused on the main post-translational modifier of Nrf2, the Keap1-Cul3 ubiquitination axis. Keap1 is highly abundant in cysteine residues, an amino acid that contains highly reactive thiol groups (-SH). When ROS levels increase, neighboring thiol groups of Keap1 get oxidized by the highly nucleophilic electrons present on oxygen radicals, and interact with one-another to form disulfide bonds (R-**S-O-S**-R), leading to conformational and functional changes in Keap1 [87]. It is for this reason that the Keap1-Nrf2 interaction is especially sensitive to ROS, which we have shown to be induced during early as well as later stages of *de novo* KSHV infection of HMVEC-d cells [16].

To determine if ROS are responsible for the induction of tNrf2 by KSHV, we utilized N-Acetylcysteine (NAC) and pyrrolidine dithiocarbamate (PDTC), two well-characterized anti-oxidants. At 2 hr p.i., cells pretreated with NAC exhibited decreased pNrf2 and tNrf2 induction by 82% and 77%, respectively (Fig. 4B). Similarly, PDTC pretreatment inhibited pNrf2 and tNrf2 induction by 87% and 96%, respectively (Fig. 4B). Because NAC and PDTC can affect cells in ROS-independent ways, as in the case of their inhibition of NF-κB, we used an additional ROS inhibitor, Diphenyleneiodonium (DPI), which has been shown to inhibit ROS in macrophages and endothelial cells [88], [89]. Similarly to NAC and PDTC, DPI treatment of uninfected HMVEC-d cells reduced, albeit weakly, the levels of pNrf2 (Fig. 4C, lanes 1–6), likely by decreasing basal ROS activity. More importantly, DPI pretreatment significantly reduced KSHV-mediated tNrf2 and pNrf2 induction in a dose-dependent manner (Fig. 4C, lanes 7–12), while not affecting KSHV-mediated NF-κB activation (Fig. S2B). These results further confirmed the importance of ROS induction in Nrf2 activation.

At the later time points (24 hr p.i.), cells pretreated with NAC exhibited diminished pNrf2 and tNrf2 induction by 83% and 77%, respectively (Fig. S2C, lane 3). PDTC pretreatment inhibited pNrf2 and tNrf2 induction by 93% and 88%, respectively (Fig. S2C, lane 4). Both drugs decreased tNrf2 levels below basal levels when compared to uninfected/untreated cells, which is most likely due to their anti-oxidative properties decreasing ROS levels of the cells below basal, an effect which increases Keap1 activity and Nrf2 degradation (Fig. 2SC, compare lanes 3 and 4 to 1). Such findings were corroborated by IFA analysis, which showed that the KSHV-mediated pNrf2 nuclear localization at 2 and 24 hr p.i. was substantially abrogated by pretreatment with NAC and PDTC (Fig. S2D). However, we have shown previously that pretreatment of the cells with anti-oxidants drastically reduces the entry and infectivity of KSHV [16]. Therefore, the inhibition of Nrf2 induction observed at 24 hr p.i. by NAC or PDTC (Fig. S2C, left) may be a result of reduced KSHV infection and not necessarily due to the dependence of this induction on ROS. To address this potential confounding variable, we performed a similar experiment where NAC or PDTC were not added to the cells until 16 hr p.i., a time when latency has already been established. Eight hours after NAC or PDTC addition (24 hr p.i.), the cells were immunoblotted for Nrf2 activity. NAC treatment of the latently-infected cells inhibited tNrf2 and pNrf2 induction by 62% and 46%, respectively, while PDTC inhibited their induction by 23% and 56%, respectively (Fig. S2C, lanes 5–8). These results clearly demonstrated the importance of ROS elevation in Nrf2 activation during early KSHV latency (Fig. S2C, compare lanes 7 and 8 to 5). It is important to note that there was still some induction of pNrf2 and tNrf2 above the basal levels in uninfected cells, which suggested additional, ROS-independent mechanisms of induction at this time point.

Taken together, these results demonstrated that KSHV-mediated ROS induction is essential for Nrf2 upregulation during the early stages of infection, and although ROS are important for latent Nrf2 induction, additional pathways are likely involved.

### KSHV induces Nrf2 stabilization by modulating the Nrf2-Keap1 interaction and decreasing its ubiquitination

Since ROS were involved in KSHV-mediated Nrf2 upregulation, we next determined the effect of KSHV infection on Keap1, the centerpiece of the ROS-dependent Nrf2-ubiquitination machinery. As expected, KSHV infection induced tNrf2 and pNrf2 (Fig. 4D, top two panels). Simultaneously, we observed a decrease in Keap1 levels to 0.8 and 0.3-fold at 0.5 and 2 hr p.i. when compared to uninfected cells (Fig. 4D, third panel, lanes 1–3). The levels of Keap1 were restored to that of uninfected cells at 24 hr p.i. (Fig. 4D, third panel, lanes 4–5) and real-time RT-PCR analysis suggested that this could be due to increased levels of Keap1 mRNA (Fig. 4E). This positive regulatory feedback loop that exists between Nrf2 and the Keap1 gene promoter has been previously described [90].

Although a decrease in absolute Keap1 levels is a strong indicator of its decreased inhibitory activity on Nrf2, we wanted to determine how *de novo* KSHV infection affects Nrf2-Keap1 interaction *per se*. According to the “open-closed conformation” model (Fig. S1), if KSHV disrupts Keap1-Cul3 cycling, we would not expect an increase in the interaction levels between Nrf2 and Keap1 despite an increase in total Nrf2 levels, because Keap1 molecules are complexed with already ubiquitinated Nrf2. To assess this, we performed co-immunoprecipitation (co-IP) studies by pulling down with anti-Keap1 antibody and Western blotted for Nrf2. When normalizing to whole cell lysate (WCL) β-tubulin, we observed a decrease in co-IPed Nrf2 by 13, 40 and 2% at 0.5, 2 and 24 hr p.i., (Fig. 4F, first and second panel, and quantification in Fig. 4G, top graph-black bars). Moreover, when accounting for robust WCL Nrf2 induction, the relative interaction between Keap1-Nrf2 decreased 65%, 84% and 43% at 0.5, 2 and 24 hr p.i., respectively (Fig. 4G, top graph-white bars). Such findings demonstrated that new Nrf2 is being generated and is not being bound and targeted by the Keap1-Cul3 inhibitory axis.

To further demonstrate that these events lead to decreased Nrf2 degradation, we determined the levels of lysine-48 (K-48) ubiquitination of Nrf2, a marker for its proteasomal degradation, and compared to the overall induction of Nrf2 in the WCL. As expected, when normalized to β-tubulin, the ubiquitination levels of Nrf2 increased slightly by 30, 24 and 10% at 0.5, 2 and 24 hr p.i., respectively (Fig. 4F, third panel, and Fig. 4G, bottom graph-black bars), likely due to the increased amount of Nrf2 pulled down (Fig. 4F, fourth panel). As a consequence, when we normalized to the total Nrf2 pulled down, relative K-48 ubiquitination levels decreased by 48, 68 and 39% at the same time points (Fig. 4G, bottom graph-white bars), indicating that although KSHV infection results in a slight increase in Nrf2-Keap1 pulled down, a lesser fraction of it is in the ubiquitinated form.

Collectively, these Nrf2-Keap1 pulldown experiments suggested that KSHV infection likely strengthens the already existent, ubiquitinated Nrf2 interaction with Keap1, resulting in Keap1 saturation and possible degradation, while concomitantly allowing the newly-translated Nrf2 to rapidly accumulate in the non-ubiquitinated and active form.

### KSHV binding is important for early Nrf2 induction

**Figure 5:**
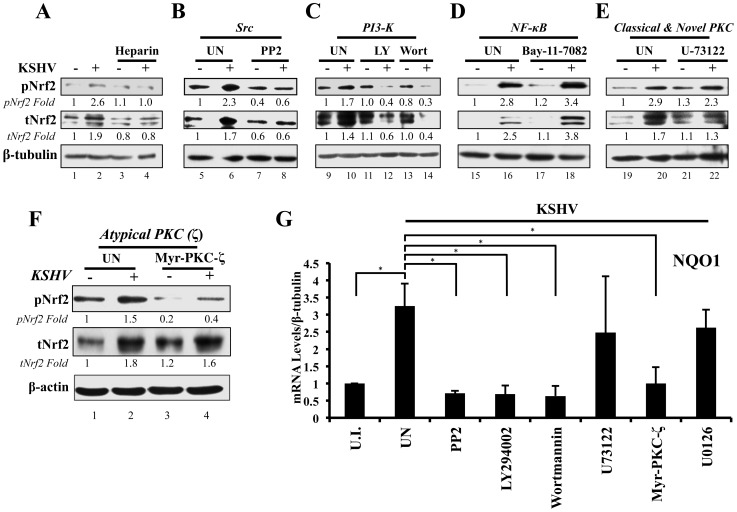
Inhibition of virus binding, Src, PI3-K and PKC-ζ and their effect on KSHV-mediated Nrf2 induction. **A**) Starved HMVEC-d cells were infected with either functional KSHV (lane 2) or with heparin-treated (20 µg/ml for 1 hr) KSHV (lane 3). Media alone (lane 1) or 20 µg/ml of heparin alone (lane 4) were used to determine if heparin had any effect on Nrf2 activity. **B–E**) HMVEC-d cells were starved for 8 hr, during which time they were treated with Src inhibitor PP2 (**B**), PI3-K inhibitors LY294002 (irreversible) and Wortmannin (reversible) (**C**), NFκB inihibitor Bay-11-7082 (**D**), PLC-γ inhibitor U-73122 (**E**), and Myristoylated PKC-ζ pseudosubstrate to inhibit PKC-ζ (consists of amino acids 113–129 of the substrate region) (**F**). One hour after drug or mock (DMSO) treatment, cells were infected for 30 min with KSHV (20 DNA copies/cell). Protein lysates were immunoblotted for pNrf2 and tNrf2, while β-tubulin/β-actin was used as a loading control. Fold inductions normalized to β-tubulin/β-actin and relative to the uninfected (U.I.) condition (arbitrarily set to 1) are indicated. **G**) HMVEC-d cells were treated with inhibitors as in panels B–F, infected with KSHV (20 DNA copies/cell) for 4 hr to allow Nrf2-dependent gene transcription to take place, and subjected to real-time RT-PCR for the Nrf2 target gene NQO1 [NAD(P)H quinone oxidase 1]. Bars indicate the fold change compared to the uninfected/untreated condition (arbitrarily set to 1) ± SD for 3 independent replicates. * = p<0.05.

To determine if KSHV interaction with its cell-surface binding and entry receptors is necessary for Nrf2 activation, we incubated KSHV with heparin, which interacts with the virus envelope glycoproteins and prevents their interaction with heparan sulfate, thereby blocking KSHV-mediated binding and associated signaling [7]–[9], [11]. While infection with KSHV for 2 hr induced tNrf2 and pNrf2 by 1.9 and 2.6-fold, respectively, infection with heparin-treated KSHV or heparin alone did not induce any significant changes in Nrf2 (Fig. 5A). In addition, although pNrf2 accumulated in the nuclei of HMVEC-d cells during KSHV infection, heparin-treated KSHV and heparin alone did not induce nuclear accumulation of pNrf2 when observed by IFA (Fig. S3). These results demonstrated that KSHV interaction with the cell-surface receptors is essential in inducing the observed Nrf2 activity early during *de novo* infection, and provided additional verification of the specificity of KSHV-dependent Nrf2 induction.

### KSHV-induced early signaling is essential for Nrf2 activation

*De novo* KSHV infection initiates a signaling cascade that induces many kinases [6]. Induction of FAK, Src, PI3-K/Akt, Rho-GTPases, EphA2 and ROS is important for KSHV macropinocytosis and entry, whereas induction of PKC-ζ, ERK1/2 and NF-κB is required for proper establishment of viral gene expression [14]–[21]. Because we observed increased Ser-40 phosphorylation of Nrf2 beyond the constitutive phosphorylation induced by H_2_O_2_ (Fig. 2), we hypothesized that one or more of these kinases induced by KSHV could induce this phosphorylation. Interestingly, atypical PKCs, PI3-K, ERK1/2 and Src have also been reported to induce Nrf2 phosphorylation in various cellular models, so we decided to assess the effect of their inhibition on Nrf2 phosphorylation during KSHV infection [67]–[72], [91]–[93]. To determine this, we pretreated cells with specific kinase inhibitors for 1 hr prior to infection with KSHV for 30 min, at which point the cells were Western blotted for tNrf2 and pNrf2 levels.

#### Src inhibition

While KSHV infection of untreated HMVEC-d cells induced tNrf2 and pNrf2 by 1.7 and 2.3-fold, respectively, the Src-specific inhibitor PP2 abolished such activation (Fig. 5B). Moreover, PP2 treatment also decreased the basal levels of pNrf2 and tNrf2 in uninfected cells (Fig. 5B), implicating Src in constitutive Nrf2 phosphorylation and stability.

#### PI3-K inhibition

LY294002, a specific covalent (irreversible) inhibitor, and Wortmannin, a specific non-covalent (reversible) inhibitor, were used to assess the importance of PI3-K on Nrf2 induction by KSHV (Fig. 5C). Interestingly, in the absence of KSHV, neither drug affected the basal levels of Nrf2 activity, arguing against a role for PI3-K in constitutive Nrf2 activity in HMVEC-d cells (Fig. 5C, compare lanes 9, 11 and 13). However, when infection was carried out in the presence of either LY294002 or Wortmannin, tNrf2 levels decreased by 40% and 60%,respectively, while pNrf2 decreased by 60% and 70%, respectively, even when compared with uninfected/untreated cells (Fig. 5C, compare lanes 12 and 14 with 10). This is an interesting finding, which suggested that PI3-K inhibition could be utilized to switch the effect of KSHV infection on Nrf2 from an inductive one to an inhibitory one.

#### NF-κB inhibition

The transcription factor NF-κB, a powerful pro-inflammatory agent, is induced during KSHV infection to aid in viral and host gene expression. Inhibition of NF-κB with the chemical inhibitor Bay-11-7082 did not affect the ability of KSHV to induce Nrf2 activity (Fig. 5D), excluding a role for NF-κB, or its downstream targets, in the activation of Nrf2 during the early stages of KSHV infection.

#### Classical and novel PKC inhibition

The protein kinase C (PKC) family of kinases consists of three distinct subgroups: the classical (c), novel (n) and atypical (a) PKCs. While cPKCs require both Ca^2+^ and diacylglycerol (DAG) for activation, nPKCs require only DAG for their activity [91]–[93]. In both cases, Ca^2+^ and DAG are supplied by PLC-γ, a molecule that is activated by KSHV through multiple pathways [92], [94]–[96]. To determine if cPKCs and nPKCs are involved in KSHV-mediated Nrf2 activation, we used the PLC-γ inhibitor U-73122, which would deplete the signaling pathway of DAG and Ca^2+^, effectively inhibiting cPKC and nPKC activation. KSHV was able to induce pNrf2 phosphorylation similarly in U-73122 or mock-treated cells, indicating that these PKCs are not important for Nrf2 phosphorylation during *de novo* KSHV infection (Fig. 5E, top panel). To our surprise, U-73122 seemed to reduce the induction of tNrf2 by KSHV infection (Fig. 5E, middle panel). This is most likely an off-site effect not related to the activation of cPKCs and nPKCs, but rather due to the inhibition of Ca^2+^ metabolism during KSHV infection, which likely affects proper ROS induction.

#### Atypical PKC inhibition

Unlike cPKCs and nPKCs, aPKCs do not rely on either Ca^2+^ or DAG for their activation and are not affected by U-73122 treatment [92]. Of the two members of the aPKCs, we focused on PKC-ζ since previous studies from our laboratory have established it as a key signaling agent in *de novo* KSHV infection [10]. To determine if PKC-ζ is important for Nrf2 activation, we used a specific inhibitor composed of the myristoylated form of a decoy polypeptide that corresponds to the PKC-ζ substrate region (Myr-PKC-ζ). Pretreatment of the cells with Myr-PKC-ζ fully abolished basal Nrf2 phosphorylation as well as the KSHV-mediated induction (Fig. 5F, top panel). Interestingly, Myr-PKC-ζ pretreatment did not affect KSHV-mediated induction of tNrf2 (Fig. 5F, middle panel). These results implicated PKC-ζ as an essential agent in mediating Nrf2 phosphorylation during KSHV infection while not affecting tNrf2 protein accumulation.

To determine if the kinase inhibitors that affect Nrf2 accumulation and/or Ser-40 phosphorylation also affect Nrf2 activity at the transcriptional level, we assessed the mRNA levels of NQO1, an Nrf2 target gene. As expected, KSHV infection of untreated HMVEC-d cells for 4 hr induced NQO1 expression by ∼3.5-fold (Fig. 5G). PP2, LY294002, Wortmannin and Myr-PKC-ζ pretreatment resulted in a robust inhibition of NQO1 mRNA upregulation induced by KSHV infection (Fig. 5G). U-73122 and U0126 (ERK1/2 inhibitor) did not show significant levels of NQO1 mRNA reduction (Fig. 5G), consistent with the U-73122 Western blot experiment (Fig. 5E).

These results clearly demonstrated that multiple signaling molecules induced by KSHV early during infection, including Src, PI3-K, and PKC-ζ, play an essential role in the induction of Nrf2 phosphorylation and activity.

### KSHV infection induces Nrf2 DNA-binding activity

**Figure 6:**
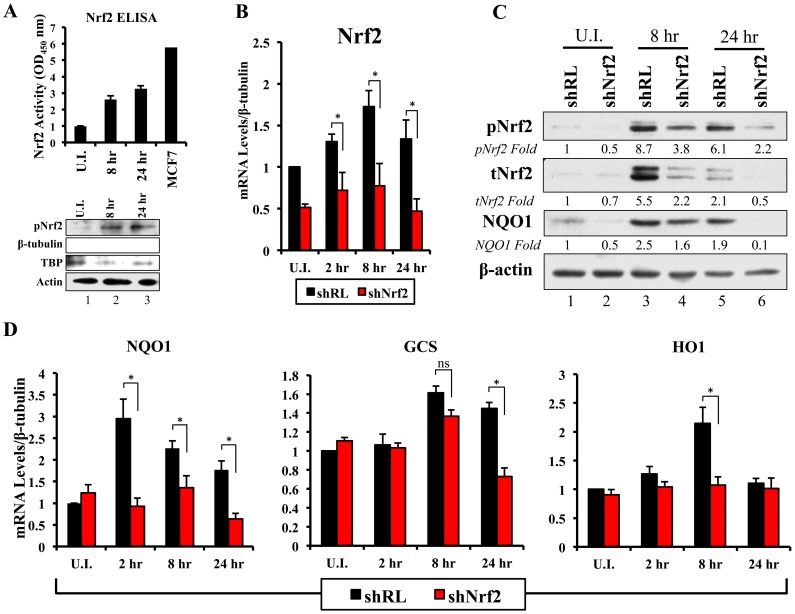
Lentiviral knockdown of Nrf2 and the effects on target genes during KSHV infection. **A**) Top: Nrf2 ELISA was used to quantify the DNA (ARE)-binding affinity of Nrf2 during KSHV infection (20 DNA copies/cell). Nuclear proteins from infected and uninfected cells were quantified using a BCA assay, and equal amounts of protein (15 µg) were assayed using the TransAM Nrf2 ELISA Kit. The OD_450_, measuring the binding of Nrf2, in the uninfected condition (U.I.) was arbitrarily set to 1, and the bars indicate mean fold induction ± SD for two independent replicates. 30 µg of MCF7 nuclear extract was used as a positive control for Nrf2 activity. Bottom: Western blot for pNrf2 in the nuclear fraction was performed for quality control. β-tubulin was used as a cytoplasmic control, TATA Binding protein (TBP) was used as a nuclear control, and β-actin was used as loading control. **B**) Real-time RT-PCR analysis assessing Nrf2 mRNA levels in HMVEC-d cells transduced with lentiviral vectors expressing short-hairpin RNA against Renilla luciferase (shRL – control) or against Nrf2 (shNrf2) for at least 24 hr. The uninfected (U.I.) shRL condition was arbitrarily set to 1 and the bars indicate mean fold induction ± SD for 4 independent experiments. **C**) Representative Western blot analysis assessing Nrf2 knockdown in HMVEC-d cells transduced with lentiviral vectors expressing shRL or shNrf2 for at least 24 hr, and infected with KSHV (20 DNA copies/cell) for the indicated time points. NQO1 induction was used to determine the activity of Nrf2 transcriptional activity during KSHV infection. β-actin was used as a loading control. **D**) Real-time RT-PCR analysis of Nrf2 target genes involved in ROS homeostasis. β-tubulin was used as an endogenous control, the U.I. shRL cells were arbitrarily set to 1, and the bars indicate mean fold induction ± SD for 4 independent experiments. * = p<0.05. (NQO1 = NAD(P)H quinone oxidase 1; GCS = Gamma-glutamylcysteine synthase; HO1 = Heme oxygenase 1).

To assess if the observed KSHV-mediated Nrf2 accumulation, phosphorylation, and nuclear accumulation was transcriptionally active, we first assessed the DNA-binding activity of Nrf2. To assess this, we isolated nuclear protein from HMVEC-d cells infected for 8 and 24 hr and performed an Nrf2 ELISA assay. The Nrf2 ELISA assay measures the binding activity of transcriptionally active Nrf2 to the ARE sequence located on specific oligonucleotides immobilized at the bottom of each well. Upon addition of equal amounts of nuclear lysate per condition, calorimetric measurements provide a relative measure of the binding activity of Nrf2 in each sample, which increases linearly with increased Nrf2 binding affinity. As expected, infection with KSHV induced Nrf2 DNA-binding activity by ∼3-fold at 8 and 24 hr p.i. when compared to uninfected cells (Fig. 6A, top panel). A Western blot of the lysates is shown for quality control (Fig. 6A, lower panel).

### Lentiviral transduction with shNrf2 abolishes KSHV-mediated Nrf2 target gene induction

Nrf2 transcriptionally activates genes involved in a variety of functions such as ROS homeostasis, apoptosis, cell migration, angiogenesis and drug resistance. KSHV-mediated Nrf2 induction may prime the cell and provide an environment conducive to KSHV infection, especially considering that several Nrf2-target genes are induced during *de novo* KSHV infection. To determine what particular host genes KSHV infection induces in an Nrf2-dependent manner, we created HMVEC-d cells deficient in Nrf2 by transducing them with lentiviral vectors expressing either short hairpin RNA against the Renilla luciferase mRNA (shRL–control) or short hairpin RNA against Nrf2 mRNA (shNrf2). These cells were then infected with KSHV for various time points and the level of Nrf2 activity was assessed for each condition. To assess the efficiency of the knockdown, we performed real-time RT-PCR analysis using Nrf2-specific primers. We observed a consistent ∼50–60% reduction in Nrf2 mRNA in shNrf2 cells compared to shRL cells during the whole course of infection (Fig. 6B). To further verify the knockdown efficiency, Western blot analysis was carried out to determine the protein levels of Nrf2. We observed a drastic reduction in Nrf2 protein levels in shNrf2 cells compared to shRL cells (Fig. S4A). To assess the specificity of the knockdown, we determined the levels of ERK1/2 and β-actin, which did not reveal any significant variation between the conditions (Fig. S4A).

Next, we determined the effect of the knockdown on the induction of several well-characterized Nrf2 target genes involved in ROS homeostasis. NAD(P)H quinone oxidase 1 (NQO1) is an important anti-oxidative agent involved in the clearance of quinones and hydroquinones, and is considered a reliable reporter of Nrf2 transcriptional activity [34], [45], [87]. Western blot analysis revealed that KSHV infection of shRL cells induced NQO1 levels by 2.5 and 1.9-fold at 8 and 24 hr p.i., respectively (Fig. 6C, third panel). Infection of shNrf2 cells, on the other hand, revealed significantly lower basal and induced NQO1 levels (Fig. 6C, third panel). These induction levels closely mirrored the induction and knockdown levels of Nrf2 as observed during the same time points (Fig. 6C, top two blots). Real-time RT-PCR analysis also showed that KSHV infection of shRL cells induced NQO1 mRNA to a significantly higher extent than during the infection of shNrf2 cells (Fig. 6D, left panel, compare black and red bars). Gamma-glutamylcysteine-synthase (GCS) is an essential component in the machinery that synthesizes glutathione, and a well-known Nrf2 target gene [34], [42], [87]. KSHV infection of shRL and shNrf2 cells induced GCS mRNA at 8 hr p.i. by 1.6 and 1.4-fold, respectively, showing that Nrf2 induction was not important for KSHV-mediated GCS upregulation at this time of infection (Fig. 6D, middle panel). At 24 hr p.i., however, KSHV induced GCS expression only in shRL cells (1.5-fold), while failing to do so in shNrf2 cells, indicating that at this time point, KSHV-mediated Nrf2 activation is required for the induction of GCS (Fig. 6D, middle panel). Heme oxygenase 1 (HO1), another well-characterized Nrf2 target gene involved in heme metabolism [34], [43], [44], [87], was significantly induced by KSHV infection of shRL cells (2.1-fold) at 8 hr p.i., but not shNrf2 cells (1.1-fold) (Fig. 6D, right panel). KSHV infection failed to induce HO1 in either condition at 24 hr p.i. (Fig. 6D, right panel).

In addition to stress-related genes, we observed other genes that are induced by *de novo* KSHV infection and that have also been shown recently to be Nrf2 target genes. Specifically, we observed that KSHV infection induced the anti-apoptotic protein Bcl-2 by ∼2-fold during the course of infection of shRL cells, and such induction was abrogated in the infection of shNrf2 cells (Fig. S4B). Glucose 6-phosphate dehydrogenase (G6PD), transaldolase (TALDO) and transketolase (TKT), three important molecules essential in the pentose phosphate pathway and nucleotide synthesis (Fig. S4D), were induced by KSHV, and this induction was Nrf2-dependent (Fig. S4C), as recently shown by Yamamoto *et al* (2012) [49]. Nrf2 knockdown did not significantly alter KSHV-mediated NF-κB activation, ruling out a role for Nrf2 in this induction during *de novo* infection (Fig. S5).

Collectively, these results clearly demonstrated that KSHV-mediated Nrf2 activation induces previously-described Nrf2 target genes, and that such activation can be severely abrogated with lentiviral vectors expressing shNrf2.

### Nrf2 plays an important role in the early induction of VEGF-A and VEGF-D by KSHV

**Figure 7:**
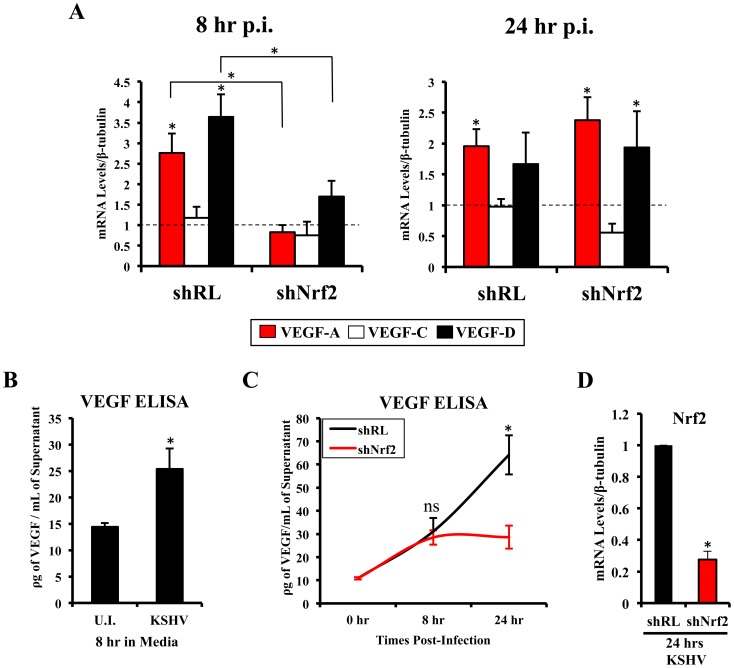
VEGF activity during KSHV infection and its dependence on Nrf2. **A**) Starved HMVEC-d cells were infected with KSHV (20 DNA copies/cell) for 8 hr (left) and 24 hr (right) prior to real-time RT-PCR analysis for 3 members of the vascular endothelial growth factor (VEGF) family: VEGF-A (red), C (white) and D (black). β-tubulin was used as an endogenous house-keeping control and gene expression of uninfected cells (not shown) was arbitrarily set to 1 as depicted by the horizontal dashed line, while the bars indicate mean fold induction ± SD for 3 independent experiments. * = p<0.05 for each time/gene when compared to the respective U.I. control. **B**) VEGF_165_ ELISA (A-isoform) (Quantikine Kit – R&D) was performed on starved HMVEC-d cells infected with KSHV (20 DNA copies/cell) for 8 hr prior to supernatant collection and quantification of absolute levels using a standard curve. **C**) VEGF_165_ ELISA was performed in shRL and shNrf2 cells infected with KSHV (20 DNA copies/cell) for the indicated times post-infection. For panels B and C, values indicate the mean of supernatant protein concentrations ± SD for 3 independent replicates. **D**) Real-time RT-PCR of Nrf2 mRNA in conditions from panel C, to determine the efficiency of lentiviral knockdown of Nrf2. * = p<0.05.

Vascular endothelial growth factor (VEGF), an angiogenic factor, plays an essential role in the formation of KSHV pathogenesis and KS histopathology along with angiogenin by mediating neovascularization and proliferation [97]–[104]. We have shown before that KSHV infection of HMVEC-d cells induces VEGF expression and secretion [104]. While certain KSHV genes have been implicated in VEGF induction, the mechanisms of VEGF induction during *de novo* infection have not been elucidated. According to recent studies, Nrf2 knockdown results in decreased angiogenesis in colonic adenocarcinoma cells and endothelial tube formation assays, both due to decreased expression of HIF-1α [52]–[54]. We examined whether Nrf2 was responsible for the induction of three important members of the VEGF family (VEGF-A, C and D). As expected, KSHV infection of shRL cells increased VEGF-A gene expression by 2.8 and 2.0-fold at 8 and 24 hr p.i., respectively (Fig. 7A, red bars). Upregulation in VEGF-A gene expression was dependent on Nrf2 at 8 hr p.i., since infection of shNrf2 cells did not show any increase at this time, but was independent of Nrf2 at 24 hr p.i. as it showed a 2.4-fold upregulation at this time (Fig. 7A, red bars). KSHV infection of shRL cells induced VEGF-C only by 1.2-fold at 8 hr p.i., and no effect (1-fold) at 24 hr p.i. (Fig. 7A, white bars). More interestingly, we identified a new VEGF, VEGF-D, which was induced by 3.6 and 1.7-fold at 8 and 24 hr p.i., respectively, in shRL cells (Fig. 7A, black bars). Only a mild induction was observed during the infection of shNrf2 cells at 8 hr p.i., but a 2-fold induction was observed 24 hr p.i., suggesting Nrf2 dependence only in the earlier stages of infection (Fig. 7A, black bars).

To determine if VEGF-A secretion levels were dependent on Nrf2 expression similarly to its gene expression, we performed a VEGF ELISA on the supernatant of infected cells. KSHV-infected HMVEC-d cells had elevated VEGF-A levels in their supernatants compared to uninfected cells at 8 hr p.i. (Fig. 7B). While infection of shRL cells exhibited a steady increase in VEGF-A supernatant levels at 8 and 24 hr p.i. (Fig. 7C, black line), infection of shNrf2 displayed only a moderate increase in VEGF-A supernatant levels (Fig. 7C, red line), further confirming the importance of Nrf2 in VEGF-A expression. To verify that the knockdown in the shRL and shNrf2 cells was successful, we performed a real-time RT-PCR analysis using Nrf2-specific primers (Fig. 7D).

These results demonstrated that KSHV-mediated Nrf2 induction early during KSHV infection plays an important role in the expression and secretion of two important members of the pro-angiogenic VEGF family. Moreover, these data also demonstrated for the first time that *de novo* KSHV infection of HMVEC-d cells induces VEGF-D expression.

### Nrf2 activity is important for COX-2 transcriptional induction by KSHV

**Figure 8:**
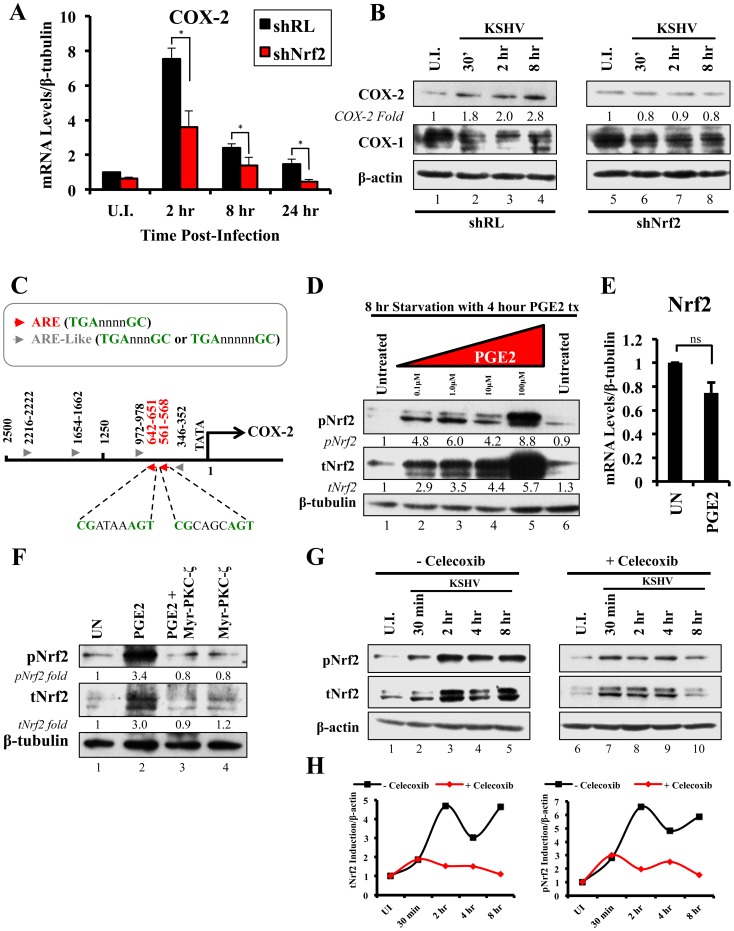
KSHV infection and the Nrf2-COX-2-PGE2 loop. **A**) HMVEC-d cells were transduced with lentiviral vectors expressing shRL or shNrf2 for 24 hr, and then infected with KSHV (20 DNA copies/cell) for the indicated time points prior to real-time RT-PCR analysis for COX-2 (Cyclooxygenase-2). The U.I. shRL condition was arbitrarily set to 1 and the bars indicate fold induction ± SD for 5 independent experiments. β-tubulin was used as an endogenous control. * = p<0.05. **B**) shRL- and shNrf2-transduced HMVEC-d cells were infected with KSHV (20 DNA copies/cell) for the indicated time points and immunoblotted for COX-2 and COX-1. β-actin was used as a loading control and the fold induction relative to each U.I. condition (arbitrarily set to 1) is indicated. **C**) The human COX-2 promoter sequence (2500 base-pairs upstream of the transcriptional start site) was obtained from ensemble.org (Accession #: ENSG00000095303). Antioxidant Response Element (ARE) consensus sequences are indicated with red arrows, whereas ARE-like sequences are identified with grey arrows. **D**) Starved HMVEC-d cells were treated with an increasing concentration (0.1–100 µM) of Prostaglandin E2 (PGE2) for 4 hr prior to immunoblot analysis for pNrf2 and tNrf2. β-tubulin was used as loading control. **E**) Starved HMVEC-d cells were treated with 10 µM PGE2 for 4 hr prior to RNA extraction and real-time RT-PCR analysis of Nrf2 gene expression levels. β-tubulin was used as an endogenous control. The uninfected condition (U.I.) was arbitrarily set to 1, and the points indicate fold induction ± SD for 3 different replicates. ns = p>0.05. **F**) Starved HMVEC-d cells were pretreated with 10 µM Myristoylated-PKC-ζ pseudosubstrate for 1 hr before addition of PGE2 for an additional 4 hr and analysis by immunoblot assay for pNrf2 and tNrf2. β-tubulin was used as a loading control and the fold induction relative to each U.I. condition (arbitrarily set to 1) is indicated. **G**) HMVEC-d cells were pretreated with mock inhibitor or Celecoxib (10 µg/ml) for 2 hr prior to KSHV infection (20 DNA copies/cell) for the indicated time points and immunoblotted for pNrf2 and tNrf2. **H**) Graphical representation of the fold induction of pNrf2 and tNrf2 levels in Celecoxib- and mock (DMSO)-treated cells. Fold induction was normalized to β-actin and is relative to their respective U.I. condition, which was arbitrarily set to 1.

We next determined the identity of additional genes that are important in KSHV biology for which Nrf2 activity is indispensable. Cyclooxygenase-2 (COX-2) is an important pro-inflammatory enzyme that catalyzes the conversion of arachidonic acid to prostaglandin H2 (PGH2), a precursor for the synthesis of several prostaglandins, including PGE2 [105]. Our earlier studies have demonstrated that KSHV induces COX-2 expression, which results in elevated levels of PGE2 formation and secretion [25], [106], [107]. These studies also showed that NFAT and CREB are two important transcription factors in inducing the COX-2 promoter, but did not exclude additional factors that may be required for COX-2 induction by KSHV [108]. To our surprise, when COX-2 mRNA was included in real-time RT-PCR analysis as a positive marker for KSHV infection, we observed that shNrf2 knockdown of the cells significantly abrogated the KSHV-mediated COX-2 induction. The induction of COX-2 in shRL cells was 7.0, 2.4 and 1.7-fold at 2, 8 and 24 hr p.i., respectively, contrasting with the modest 3.6, 1.3 and 0.5-fold induction (>50% reduction) in shNrf2 cells (Fig. 8A). Western blot analysis was consistent with the PCR data, showing a steady increase in COX-2 protein levels during the infection of shRL cells, but no observable increase in COX-2 during KSHV infection of shNrf2 cells (Fig. 8B). As expected, no obvious changes in the pattern of expression of the constitutively expressed COX-1 protein were observed (Fig. 8B).

Because Nrf2 is a transcription factor, we explored the possibility of Nrf2 playing an important role in COX-2 induction by direct binding to the COX-2 promoter. When we analyzed the sequence of the COX-2 promoter, we identified two Nrf2 binding sites (ARE-consensus sequence - TGAnnnnGC) on the template strand (Fig. 8C, red arrows). Moreover, the coding strand also provided a sequence pattern containing multiple ARE-like domains (grey arrows), which contained either an extra or missing base-pair between the required TGA and GC (TGAnnnnnGC or TGAnnnGC). These results are in concordance with results in Figs. 8A and B, and suggested that Nrf2 likely induces COX-2 expression through its transcriptional properties.

### The COX-2 product PGE2 induces Nrf2 activity

Sharma-Walia *et al* have demonstrated that KSHV-mediated COX-2 induction, either during entry or during latency, results in increased release of PGE2 [106], while Arun *et al* have demonstrated that such secretion mediates signaling in paracrine and autocrine manners through prostaglandin receptors 1–4 (EP 1–4) [97], [106]. Among such signaling pathways is PKC-ζ, which we determined to be important for Nrf2 induction during the early stages of KSHV infection (Fig. 5F) [106]. We hypothesized that PGE2 secretion during the post-entry stages of infection might induce PKC-ζ, which may then lead to sustained Nrf2 induction even post-KSHV entry. We therefore performed a Western blot analysis on the levels of Nrf2 in HMVEC-d cells that were treated with PGE2 for 4 hr, where we observed a robust, dose-dependent tNrf2 and pNrf2 induction (Fig. 8D). Real-time RT-PCR analysis showed that this induction was not mediated at the transcriptional level, as Nrf2 mRNA expression was not significantly affected by PGE2 treatment (Fig. 8E), leading us to believe that signaling pathways mediating Nrf2 stabilization and phosphorylation were involved. We, therefore, pretreated HMVEC-d cells with Myr-PKC-ζ for 1 hr prior to addition of PGE2 for an additional 4 hr. Interestingly, although PGE2 alone was able to induce tNrf2 and pNrf2 by 3.0 and 3.4-fold, respectively, Myr-PKC-ζ was able to fully abrogate such induction (Fig. 8F), establishing PKC-ζ as a key agent in PGE2-mediated Nrf2 activation.

### COX-2 and PGE2 induce Nrf2 activity during KSHV infection

Celecoxib is a specific COX-2 inhibitor that we have previously shown to abrogate PGE2 production during KSHV infection [109]. We utilized the inhibitory effects of Celecoxib to determine if endogenous COX-2 activation and PGE2 release induced by KSHV infection are important for Nrf2 activation. We, therefore, infected HMVEC-d cells in the absence or presence of Celecoxib and assessed Nrf2 levels (Fig. 8G). Infection of mock-treated cells induced Nrf2 accumulation with a pattern similar to that observed in figure 2A, with a robust steady raise until 2 hr p.i., a small dip at 4 hr p.i., and another increase by 8 hr p.i. (Fig. 8G, lanes 1–5). Interestingly, infection of Celecoxib-treated cells induced tNrf2 accumulation and its phosphorylation during the earlier stages (30 min p.i.) of infection, but this induction did not persist throughout the monitored period of 8 hr p.i. (Fig. 8G, lanes 6–10). When we plotted the fold induction of tNrf2 and pNrf2 between these two conditions, we observed no difference at 30 min p.i., but observed a >70% fold reduction in Nrf2 activation in Celecoxib-treated cells compared to mock-treated cells from 2–8 hr p.i. (Fig. 8H).

Collectively, these results demonstrated that KSHV-induced COX-2 and PGE2 autocrine/paracrine signaling activate Nrf2 during the post-entry stages of *de novo* infection of HMVEC-d cells through induction of a signaling cascade that requires PKC-ζ.

### KSHV latent gene vFLIP is important for Nrf2 induction

**Figure 9:**
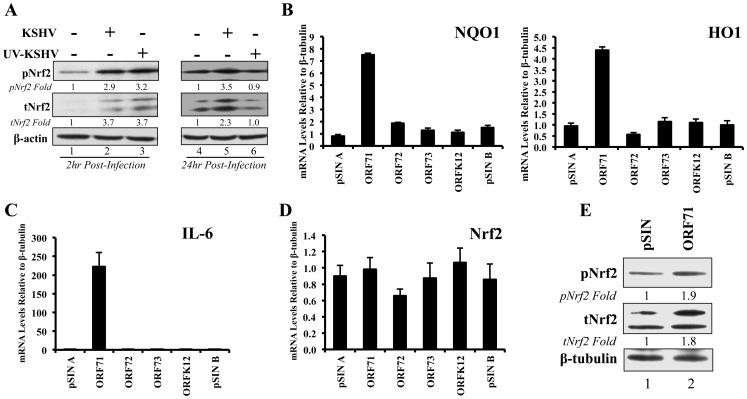
Nrf2 induction during UV-KSHV infection and during latent KSHV gene vFLIP overexpression. **A**) Starved HMVEC-d cells were left uninfected, infected with functional KSHV, or infected with UV-treated KSHV for 2 hr (lanes 1–3) and 24 hr (lanes 4–6), and immunoblotted for pNrf2 and tNrf2. β-actin was used as loading control. Fold inductions normalized to β-actin and relative to the uninfected (U.I.) condition (arbitrarily set to 1) are indicated. **B–D**) HMVEC-d cells were transduced for 72 hr using vectors containing the four latent KSHV genes (ORF71/vFLIP, ORF72/vCyclin, ORF73/LANA-1 and ORFK12/Kaposin) and the level of relevant genes were determined by real-time RT-PCR. Bars indicate fold induction relative to pSIN A (arbitrarily set to 1) ± SD for 3 independent replicates. **E**) HEK293T cells transfected with control vector or with vector containing ORF71/vFLIP for 24 hr were assessed for levels of tNrf2 and pNrf2.

Because COX-2 is also induced by KSHV latency, we wanted to determine if latent gene expression is important for Nrf2 induction during the later stages of *de novo* infection. Ultraviolet (UV) light treatment of KSHV creates thymidine dimers between adjacent thymine residues of its DNA, and this abolishes its ability to replicate or properly express its genome. This process, however, does not affect the envelope and capsid of the virion, creating a virus that is incapable of establishing proper latent infection, but still capable of inducing early, gene expression-independent events propagated by virion-host interaction [24]. At 2 hr p.i., UV-KSHV induced tNrf2 and pNrf2 by 3.7 and 3.2-fold, respectively, and this was comparable to the induction observed by the untreated virus (Fig. 9A, lanes 1–3). However, we did not observe any induction in Nrf2 activity 24 hr p.i. with UV-KSHV, in contrast to such activation with untreated virus (Fig. 9A, lanes 4–6). IFA analysis also showed that while UV-KSHV could induce nuclear accumulation of pNrf2 at 2 hr p.i., no such effects were seen at 24 hr p.i. (Fig. S6A). These studies demonstrated that KSHV binding to HMVEC-d cell-surface receptors is essential in inducing Nrf2 activity during the early stages of *de novo* KSHV infection, while expression of latent genes was involved in Nrf2 induction during the late stages of infection.

To assess which KSHV latent gene(s) was responsible for the observed Nrf2 induction, HMVEC-d cells were transduced with lentiviruses expressing four known latent KSHV genes (ORFs 71, 72, 73 and K12) along with two vector controls (pSIN A and pSIN B). Real-time RT- PCR analysis revealed that all latent genes were successfully expressed in their respective transductions (Fig. S6B). Of the four KSHV genes, only transduction with ORF71 (vFLIP) significantly upregulated NQO1 and HO1 mRNA expression, suggesting that vFLIP might be an important inducer of Nrf2 activity during later stages of infection (Fig. 9B). We also observed significant IL-6 upregulation by vFLIP, further verifying the proper activity and specificity of vFLIP expression given its well-known inducing effects on NF-κB (Fig. 9C). Real-time RT-PCR assessment of Nrf2 transcript did not show any significant changes during vFLIP expression (Fig. 9D), indicating that vFLIP induces the Nrf2 axis through non-transcriptional pathways. Moreover, HEK 293T cells transfected with ORF71 showed increased tNrf2 and pNrf2 levels as measured by Western blot analysis (Fig. 9E).

These studies demonstrated that expression of latent genes was involved in Nrf2 induction, and that ORF71 (vFLIP) could be one of the agents responsible for such induction.

### KSHV-infected HMVEC-d cells have enhanced intracellular pNrf2 during latency

Results described above in Fig. 9 demonstrated that latent KSHV induces Nrf2 activity in the whole population of infected cells. At the single-cell level, vFLIP expression may affect Nrf2 activity either i) by inducing intracellular signaling pathways that affect Nrf2 activity within the same cell or ii) by inducing secretion of cytokines (PGE2) and other signaling ligands, which, through autocrine and paracrine signaling, can induce Nrf2 activity in adjacent cells. In the first scenario, only infected cells would exhibit enhanced Nrf2 activity, whereas in the later, both infected and adjacent uninfected cells would exhibit enhanced Nrf2 levels. To differentiate between these two possibilities, we performed an IFA on HMVEC-d cells infected with 5-bromo-2-deoxyuridine (BrdU)-labeled KSHV. Such a technique allowed us to determine precisely which cells were infected with KSHV by staining with an anti-BrdU antibody, while simultaneously observing pNrf2 activity by staining with anti-pNrf2 antibody.

**Figure 10:**
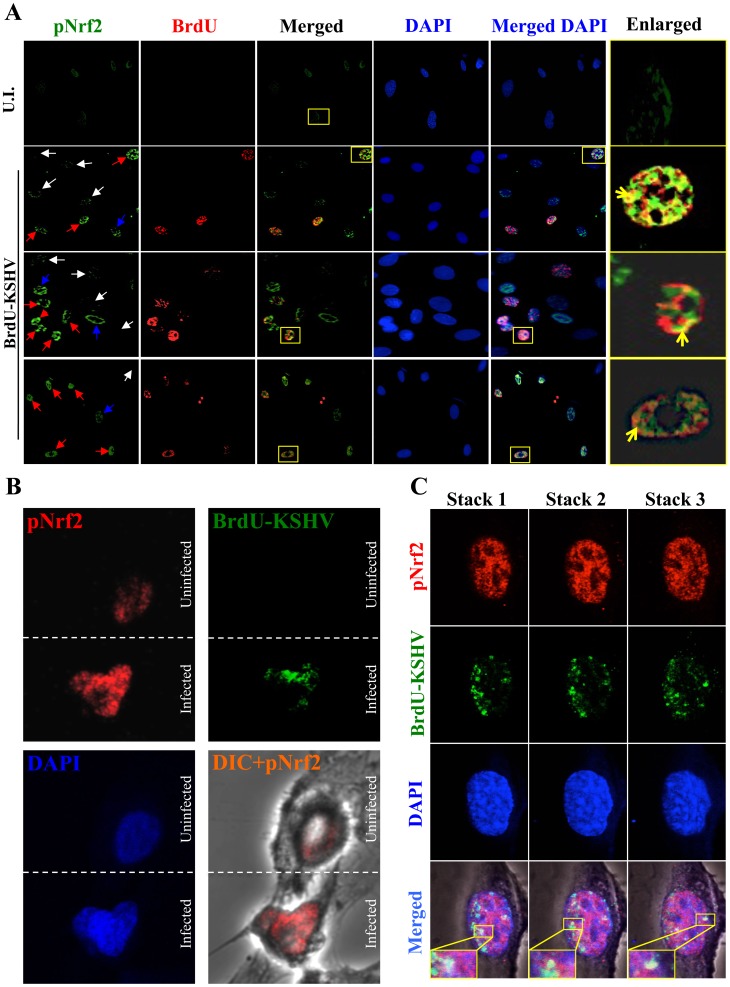
Effect of KSHV binding and gene expression on virus-mediated Nrf2 induction. **A**) Deconvoluted IFA imaging of HMVEC-d cells infected with BrdU-labeled genome containing KSHV for 24 hr, and stained with rabbit anti-pNrf2 primary antibody and goat anti-rabbit (Alexa-Fluor 488-green) secondary antibody, and with mouse anti-BrdU primary antibody and goat anti-mouse (Alexa-Fluor 594-red) secondary antibody. Yellow square = enlarged area; red arrows = infected cell; white arrows = uninfected cells with low pNrf2; blue arrows = uninfected cell with high nuclear pNrf2; blue staining = DAPI. Yellow arrows = colocalization of pNrf2 and BrdU-labeled KSHV genome. **B**) Confocal imaging of two HMVEC-d cells for pNrf2, BrdU-labeled KSHV genome and DAPI. Dashed white line separates the field in two, the top field containing an uninfected cell while the bottom field containing a 24 hr KSHV-infected cell. **C**) Confocal imaging on three separate stacks of the same cell infected with BrdU-labeled KSHV for 24 hr. Triple-merged figures are shown on the bottom-most row, and representative colocalization figures are enlarged in the yellow boxes.

As expected, the cells in the uninfected condition (negative control) showed no BrdU staining and low levels of pNrf2 in their nuclei (Fig. 10A, top row). In contrast, cells in the BrdU-KSHV condition showed significant BrdU staining (Fig. 10A, bottom 3 rows, second column). Colocalization of pNrf2 with BrdU-labeled KSHV genome was readily observed in the infected cell nuclei (Fig. 10A, bottom 3 rows, enlarged box, yellow arrows). More interestingly, in the infected condition, all the cells (14/14 or 100%) that stained for BrdU-KSHV also demonstrated high levels of pNrf2 in their nuclei (Fig. 10A, bottom 3 rows, first column, red arrows). Most uninfected cells (9/13 or ∼70%) showed very low to undetectable levels of pNrf2 staining (white arrows). However, a minority of uninfected cells (4/13 or ∼30%) showed some increase in pNrf2 activity (blue arrows), and these cells were often adjacent to the infected cells, possibly influenced by their paracrine effect. We confirmed that BrdU-KSHV infection induces pNrf2 in the nuclei of the cells through confocal imaging, which provides better resolution. Specifically, two cells, one positive and one negative for BrdU-KSHV staining, were visualized within the same field (Fig. 10B). Corroborating the IFA data, the infected cell exhibited substantially elevated levels of pNrf2 in the nucleus compared to the adjacent uninfected cell (Fig. 10B).

These results demonstrated that viral gene expression plays an important role in inducing pNrf2 levels within the infected cell, while also suggesting that an autocrine/paracrine pathway aids in this induction.

### KSHV genome and LANA-1 colocalize with pNrf2 in the infected cell nuclei

Using IFA we observed substantial colocalization of BrdU-KSHV and pNrf2 in the nuclei of infected cells (Fig. 10A, right-most column, yellow arrows). Because of the low resolution provided by IFA, we confirmed the findings using confocal microscopy, which also revealed multiple colocalization spots between the BrdU-KSHV genome and pNrf2 (Fig. 10C, merged, yellow squares).

**Figure 11:**
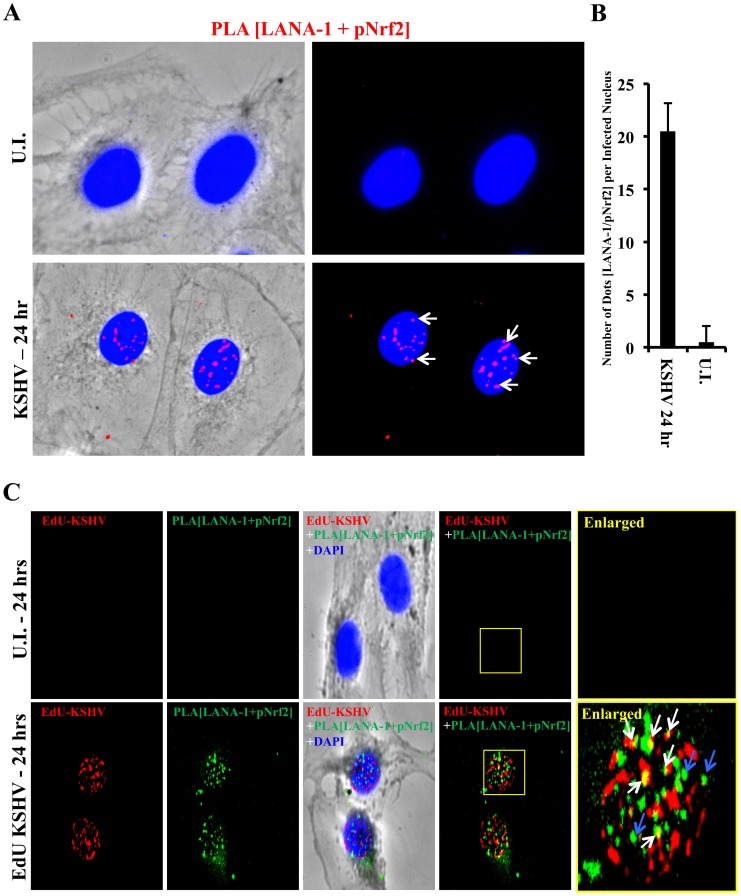
Colocalization of pNrf2 with KSHV genome and LANA-1. **A**) Proximity ligation assay (PLA) on uninfected (U.I.) and KSHV-infected cells (20 DNA copies/cell) for 24 hr. Cells were incubated for 1 hr with antibodies against pNrf2 (rabbit) and LANA-1 (mouse monoclonal), washed, incubated for 1 hr with species-specific PLA probes and 2 additional oligonucleotides to facilitate the hybridization only in close proximity (<16 nm). A ligase was then added to join the two hybridized oligonucleotides to form a closed circle and initiate a rolling-circle amplification using the ligated circle as a template after adding an amplification solution to generate a concatemeric product extending from the oligonucleotide arm of the PLA probe. Lastly, a detection solution consisting of fluorescently-labeled oligonucleotides was added, and the labeled oligonucleotides were hybridized to the concatemeric products. The signal was detected as distinct fluorescent dots. Negative controls consisted of samples treated as described but with only secondary antibodies. Confocal microscopy was used for imaging. Red dots represent LANA-1 and pNrf2; blue staining = DAPI; white arrow = PLA dot [LANA-1+pNrf2] interaction. **B**) Quantification of the number of dots in the nuclei of infected HMVEC-d cells were obtained from 3 independent, representative fields, containing ∼30 cells each. **C**) HMVEC-d cells were infected with EdU-labeled KSHV and PLA for pNrf2 and LANA-1 (green dots) was performed as described in Figure 11A prior to staining for EdU-KSHV (red). White arrows indicate the yellow colocalization spots between LANA-1+pNrf2 (PLA green spots) and EdU-KSHV genome (red). Blue arrows indicate the LANA-1+pNrf2 (PLA red spots) not colocalizing with EdU-KSHV genome.

We further performed a proximity ligation assay (PLA) between pNrf2 and LANA-1, a technique that can identify interactions between proteins that are <16 nm apart. Compared to uninfected cells, which showed essentially no PLA staining, HMVEC-d cells infected with KSHV for 24 hr showed significant staining (Fig. 11A, white arrows). When quantified, we observed ∼20 dots/nucleus in infected cells compared to the non-specific single dot/nucleus observed in uninfected cells (Fig. 11B), suggesting significant colocalization between LANA-1 and pNrf2.

We next determined whether pNrf2 and LANA-1 colocalized together on the KSHV genome or elsewhere in the nucleus, where they may affect gene expression. To determine possible triple-colocalization we performed PLA for pNrf2 and LANA-1 (green dots) and stained the EdU-labeled virus (red) (Fig. 11C). Using the BrdU-labeled KSHV was not feasible for this experiment because the IFA procedure for BrdU labeling requires DNA denaturation, a process that interferes with the PLA procedure. Interestingly, we observed that about half of the pNrf2-LANA-1 PLA puncta colocalized with the EdU-KSHV genome (Fig. 11C, bottom panel, white arrows), suggesting a possible interaction of all these three agents. In addition, we also observed the pNrf2 and LANA-1 PLA spots in areas of the nucleus where no EdU-KSHV genome was detected (Fig. 11C, bottom panel, blue arrows) which could be in part representing a complex potentially involved in the modulation of host gene expression.

These results suggested that Nrf2 interacts with the KSHV genome by utilizing one of its major regulatory proteins, LANA-1, as an intermediary.

### Nrf2 modulation does not affect KSHV entry

**Figure 12:**
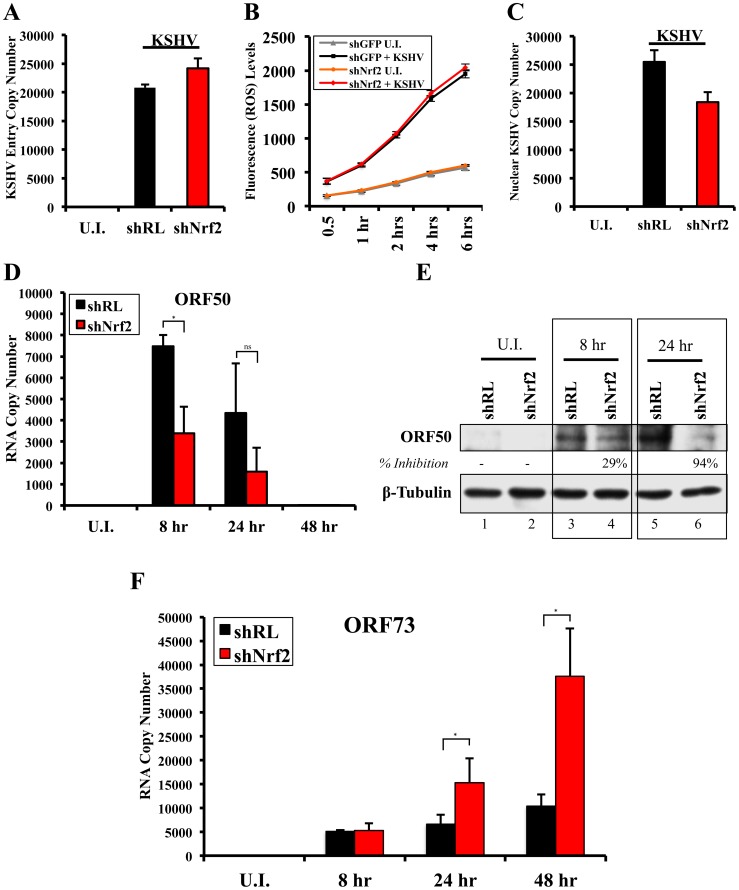
Effect of Nrf2 modulation on KSHV biology. **A**) KSHV entry assay was performed on cells transduced with shRL or shNrf2 and infected with KSHV (20 DNA copies/cell). DNA real-time PCR was performed with ORF73 gene-specific primers, and the absolute KSHV copy number was calculated from a standard curve obtained by real-time PCR of standards with known concentration of ORF73. **B**) Starved HMVEC-d cells in a 48-well plate previously transduced with lentivirus vectors expressing either shGFP or shNrf2 were labeled with the ROS-measuring dye, CM-H_2_DCFDA, and then infected with KSHV (40 DNA copies/cell) for the indicated time points prior to fluorescence measurement. Values indicate the mean ± SD for 3 independent replicates. **C**) KSHV nuclear delivery assay was performed on cells that were transduced with shRL or shNrf2 prior to infection with KSHV for 2 hr. Real-time PCR was performed using ORF73 gene-specific primers on DNA extracted from the nuclei of infected cells to determine the levels of viral DNA. The absolute copy number was calculated from a standard curve obtained by real-time PCR of standards with known concentrations of ORF73. Bars indicate mean ± SD for 3 independent replicates. **D and E**) Starved HMVEC-d cells transduced with either shRL or shNrf2 were infected for various times with KSHV (50 DNA copies/cell) and analyzed by one-step real-time PCR reaction and by WB using ORF50-specific primers and antibody, respectively. **F**) ORF73 (LANA-1) gene-specific primers were used to determine the expression levels of ORF73 from RNA as in panel 12D. The absolute copy number was calculated from a standard curve obtained by real-time PCR of RNA standards of ORF73 or ORF50 with known concentrations. Bars indicate mean copy number ± SD of 3 independent replicates. * = p<0.05, ns = p>0.05.

Since Nrf2 associated with the KSHV genome, we wanted to determine its effects on KSHV biology. We first performed a KSHV entry assay on infected shNrf2 and shRL cells and observed that shNrf2-mediated knockdown did not affect the entry levels of KSHV (Fig. 12A). Real-time RT-PCR analysis of Nrf2 mRNA demonstrated that the lentiviral knockdown of shNrf2 cells was successful (Fig. S7A). We also performed the entry assay using chemicals that have been shown in multiple systems to affect Nrf2 activity. Trigonelline, a coffee extract alkaloid that has been shown to inhibit Nrf2 transcriptional activity by inducing its nuclear export [110], abolished NQO1 and GCS mRNA upregulation by KSHV infection as measured by real-time RT-PCR (Fig. S7C). Sulforaphane and tBHQ, two anti-oxidants and well-characterized inducers of Nrf2 protein levels and activity [87], were both able to induce Nrf2 accumulation (Fig. S7D). Bay-11-7082, an NF-κB inhibitor, was included as a negative control as previous studies from our laboratory have shown that NF-κB is not involved in KSHV entry or nuclear delivery. We pretreated HMVEC-d cells with each of these chemicals for 4 hr prior to infection with KSHV for 30 min and performed a KSHV entry assay. Similar to the knockdown experiments, none of these compounds significantly affected KSHV entry (Fig. S7E), further demonstrating that Nrf2 activity does not play an important role in KSHV entry.

### Nrf2 knockdown does not affect ROS levels in HMVEC-d cells

The results of the entry experiments were surprising, as we had anticipated Nrf2 knockdown to increase KSHV entry due to increased basal ROS levels. To test whether knockdown of Nrf2 in HMVEC-d cells results in elevated ROS, we performed a ROS measurement experiment in cells transduced with shNrf2 and shGFP (shRNA against green fluorescent protein–negative control). The use of shRL-expressing lentivirus used in most of the experiments was avoided in this experiment because the vector that expresses shRL also expresses GFP (used to monitor transduction efficiency), which has the same excitation/emission spectrum as the CM-H_2_DCFDA dye, and would interfere with ROS measurement. As we have previously shown, KSHV-infected HMVEC-d cells produced 2–3-fold more ROS than uninfected cells (Fig. S7F). To our surprise, Nrf2 knockdown did not affect the basal ROS levels in uninfected cells (Fig. 12B, compare shGFP U.I. with shNrf2 U.I.). Moreover, KSHV-mediated ROS induction was unchanged between the two cell conditions (Fig. 12B, compare shGFP+KSHV with shNrf2+KSHV). Similarly, Nrf2 knockdown did not affect basal ROS production or KSHV-induced ROS even at 24 hr p.i. (Fig. S7G). Real-time RT-PCR analysis of Nrf2 mRNA demonstrated that the lentiviral knockdown of shNrf2 cells was successful (Fig. S7B).

These results demonstrated that basal Nrf2-levels are dispensable for ROS homeostasis in HMVEC-d cells and that KSHV-mediated Nrf2 induction does not affect the induction of ROS during *de novo* infection, suggesting no role for Nrf2 in KSHV entry.

### Nrf2 modulation does not affect KSHV nuclear delivery

We next determined if Nrf2 activity plays a role in KSHV nuclear delivery. We infected shNrf2 and shRL cells with KSHV, isolated the nuclei of the cells 2 hr p.i., and performed real-time DNA PCR using ORF73-specific primers. Interestingly, we observed only a slight decrease (25%) in the nuclear delivery of KSHV in shNrf2 cells compared to control cells (Fig. 12C). Real-time RT-PCR analysis of Nrf2 mRNA demonstrated that the lentiviral knockdown of shNrf2 cells was successful (Fig. S8A). We further determined the degree of nuclear delivery in cells treated with the chemicals used in Fig. S7E, which did not affect the nuclear delivery of KSHV in HMVEC-d cells (Fig. S8B). These data suggested that Nrf2 does not play an important role in KSHV nuclear delivery during *de novo* KSHV infection.

### Nrf2 knockdown decreases the early KSHV lytic gene expression burst

Our earlier studies have determined that *de novo* KSHV infection of HMVEC-d cells inevitably results in the establishment of latency [26]. Indeed, we observed a steady increase in ORF73 gene expression (Fig. S8C), whose product, LANA-1, is essential for maintenance of viral latency [26]. However, before latency is established, a transient expression of lytic genes with anti-apoptotic and immune-evasive functions peaks at the earlier stages of infection and subsides as latency takes over [26]. We observed a robust expression of ORF50, the master regulator of lytic gene expression, which decreased by 24 hr p.i. (Fig. S8D).

Since Nrf2 knockdown did not affect KSHV entry or nuclear delivery, and because Nrf2 colocalized with LANA-1 on the KSHV genome (Fig. 11), we further wanted to determine if Nrf2 plays a role in the proper establishment of KSHV latency by affecting the normal course of viral gene expression during the early stages of *de novo* infection. As expected, infection of shRL cells produced a viral expression pattern similar to that of untransduced cells, with ORF50 peaking at 8 hr p.i., declining by 24 hr p.i., and becoming essentially undetectable by 48 hr p.i. (Fig. 12D, black bars). Interestingly, ORF50 expression during the infection of shNrf2 cells was reduced by 47 and 64% at 8 and 24 hr p.i., respectively (Fig. 12D, red bars). A Western blot analysis of ORF50 expression confirmed the decreased expression of ORF50 at 8 and 24 hr p.i, by 29 and 94% respectively (Fig. 12E). We further assessed the expression pattern of additional lytic genes previously shown to be expressed during early *de novo* infection, such as ORFK8, K5 and vIRF2, and observed decreased expression during infection of shNrf2 cells compared to shRL cells, suggesting an important role for Nrf2 in the expression of the early lytic KSHV genes during *de novo* infection of HMVEC-d cells (Fig. S9).

### Nrf2 knockdown increases LANA-1 expression in infected cells

In contrast to lytic genes, ORF73 (LANA-1) gene expression was significantly upregulated by 3-fold at 24 hr p.i. and by 3.6-fold at 48 hr p.i. in shNrf2 cells compared to shRL cells (Fig. 12F). The knockdown for each condition was efficient (Fig. S8E), and KSHV infection was able to induce NQO1 gene expression in shRL cells, but not shNrf2 cells, as we previously determined (Fig. S8F).

**Figure 13:**
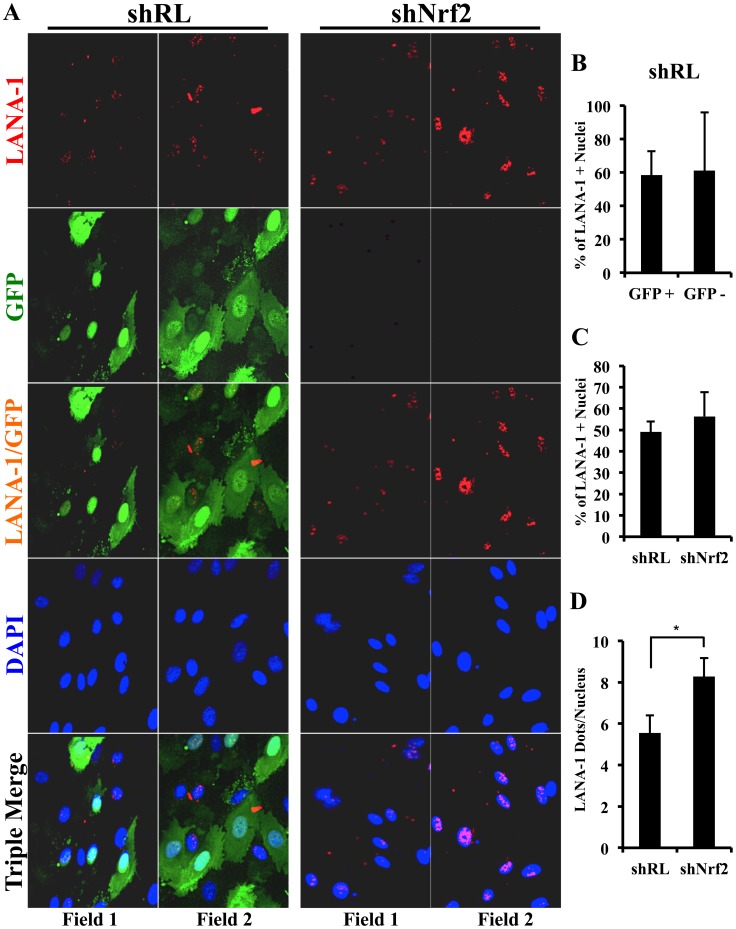
LANA-1 puncta during infection of Nrf2-deficient cells. shRL- and shNrf2-transduced HMVEC-d cells were infected with KSHV (40 DNA copies/cell) for 24 hr prior to IFA analysis using a rabbit LANA-1 specific antibody (red). The vector containing shRL expresses the green fluorescent protein (GFP), which explains the green color of shRL cells that have been successfully transduced with the vector. **B**) Quantification of the number of KSHV+ (LANA-1+) cells in the shRL-transduced cells that expressed GFP (successfully transduced) and cells that did not (unsuccessfully transduced). Bars represent mean ± SD for three individual fields containing at least 10 cells each (panel A, row 3, columns 1–2). **C**) Quantification of the number of KSHV+ (LANA-1+) cells in shRL vs. shNrf2 conditions. Bars represent mean ± SD for three individual fields containing at least 10 cells each (panel A, row 4). **D**) Quantification of the number of LANA-1 dots/nucleus in shRL vs. shNrf2 conditions. Bars represent mean ± SD for three individual fields containing at least 10 cells each (panel A, row 4).

To assess how Nrf2 knockdown increased LANA-1 expression, we performed an IFA analysis of LANA-1 during the infection of shRL and shNrf2 cells (Fig. 13A). As expected, the shRL condition consisted of cells expressing GFP, which is part of the shRL lentiviral vector used to measure transduction efficiency. In contrast, the shNrf2 lentiviral vector does not code for GFP. For the purposes of this experiment, fields from the shRL condition containing ∼50% transduction efficiency were obtained to more accurately assess if control vector (shRL) lentiviral transduction had any effects on subsequent KSHV infectivity. As observed by the quantification in Fig. 13B, the same percentage of GFP-positive cells and GFP-negative cells, ∼60%, were infected with KSHV, arguing against a possible role for the control lentiviral transduction in affecting subsequent KSHV infectivity. Furthermore, KSHV infected a similar portion of shRL cells compared to shNrf2 cells, indicating that Nrf2 knockdown does not affect KSHV infection rate (Fig. 13C). Such a finding is consistent with the entry and nuclear delivery assays in Fig. 12A and C. We then quantified the number of LANA-1 puncta per nucleus of each infected cell, and observed that shNrf2 nuclei exhibited, on average, ∼60% more LANA-1 puncta compared to infected shRL cell nuclei (Fig. 13D).

Collectively, these results suggest that Nrf2 knockdown does not affect infectivity with KSHV, but increases LANA-1 transcript expression and puncta formation within the infected cells.

## Discussion

**Figure 14:**
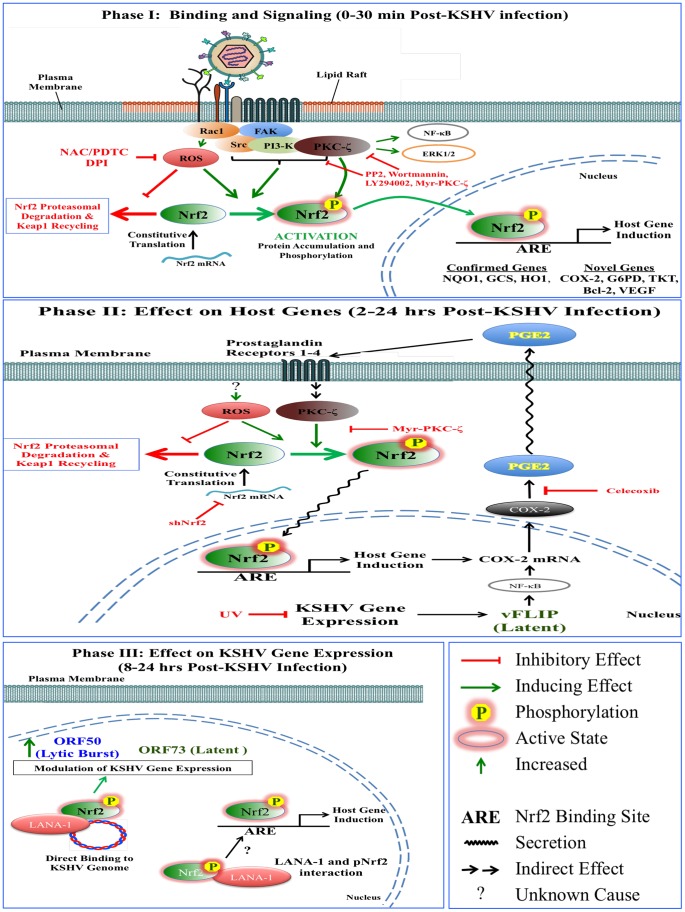
Schematic diagram showing Nrf2 induction and consequences during *de novo* KSHV infection of endothelial cells. **Phase I**. KSHV binding to cellular receptors induces signaling that involves multiple kinases and ROS. ROS induction inhibits degradation of Nrf2 by the Keap1-Cul3 ubiquitination axis, allowing for its rapid accumulation inside the cells. Signaling kinases induced by the interaction of KSHV with its cell-surface receptors to facilitate virus entry and gene expression also mediate Nrf2 phosphorylation, which serves to further stabilize Nrf2 by decreasing its association with Keap1 as well as by increasing its nuclear localization. Nrf2 nuclear localization induces expression of well-known anti-oxidative target genes as well as additional genes such as COX-2, Bcl-2 and VEGF. **Phase II**. As the original signaling wanes, the Nrf2-mediated COX-2 induction releases PGE2 in the surrounding environment, which in turn induces Nrf2 activation via autocrine and paracrine signaling pathways involving PKC-ζ. This establishes a feed-forward loop where Nrf2 activation induces COX-2 expression, which releases PGE2 and mediates further Nrf2 activation. As latency initiates, vFLIP expression further fuels the COX-2/PGE2 axis, and likely other pathways, to augment Nrf2 activity. **Phase III**. As virus establishes latency, Nrf2 activity appears to be important at several levels in its biology, as its inhibition upregulates ORF73 expression and decreases ORF50 and other lytic expression. Nrf2 also colocalizes with the major latency protein, LANA-1, as determined by PLA, and both colocalized with the KSHV genome, probably modulating the expression of viral and host genes.

KSHV is the etiological agent of KS, and the importance of oxidative stress in the development of KS pathogenesis has been shown before [83]. In this comprehensive study, we have demonstrated for the first time that *de novo* KSHV infection of endothelial cells induces the powerful transcription factor Nrf2. Moreover, our studies show that such Nrf2 upregulation is i) present in *in vivo* KS skin tissues, ii) dependent on ROS induction and signaling through Src, PI3-K and PKC-ζ, and iii) important for the expression of multiple host and viral genes involved in KSHV biology. We also show that Nrf2 induction is required for optimal COX-2 expression seen during KSHV infection, which through its enzymatic product PGE2 further induced Nrf2 activity, creating a feed-forward loop between these two molecules at later time points of infection (Fig. 14). For a better understanding, we have summarized the potential implications of the multiple roles that Nrf2 plays in KSHV biology in the following sections.

### Nrf2 stability during *de novo* KSHV infection

Although the importance of Keap1 in Nrf2 inhibition and degradation has long been known, the precise mechanism how this interaction is affected by ROS and other Nrf2 inducers was just recently elucidated. Oxidative stress, Sulforaphane and tBHQ stabilize Nrf2 not by directly dissociating it from Keap1, but by increasing the binding of existent, ubiquitinated Nrf2 with the Keap1-Cul3 ubiquitination axis, thus allowing newly synthesized Nrf2 to accumulate untargeted in the cell (Fig. S1B) [61], [62]. Kobayashi *et al* (2006) had shown that *de novo* Nrf2 protein synthesis was required for Nrf2 nuclear translocation and transcriptional activation by ROS, further validating this model [111]. Indeed, our results are in full concordance with this novel mechanism. KSHV infection of HMVEC-d cells did not significantly affect the levels of Nrf2 precipitated by Keap1 pull-down despite a robust increase in cellular Nrf2 protein levels, most of which was in an un-ubiquitinated form (Fig. 4F). This process was ROS-dependent, as inhibition of ROS with the NAC, PDTC and DPI abrogated KSHV-mediated Nrf2 induction. Collectively, these results strongly suggested that KSHV induces ROS, an event that thiolizes the cysteine residues located in the linker region of Keap1, disrupting the fluidity of the “closed-to-open” conformational cycling of the inhibitory machinery, saturating the Keap1-Cul3 system, and allowing Nrf2 to accumulate in the cytoplasm of HMVEC-d cells early during *de novo* KSHV infection.

Novel pathways that may affect Nrf2 stability are under intense scrutiny. Specifically, it has been shown that GSK-3β mediates phosphorylation of certain serine residues on the Neh6 domain of Nrf2, increasing its affinity for another E3 ubiquitin ligase, β-TrCP, which is believed to mediate Nrf2 ubiquitination and proteasomal degradation [40]. However, these studies were carried out in *in vitro* systems and the biological significance of such findings remains to be determined. Moreover, although it is well-known that during latency LANA-1 inhibits GSK-3β [112], we saw no effect of *de novo* infection on GSK-3β activity (Fig. S10), making this pathway unlikely to be involved in Nrf2 activation at the observed stage of infection.

### Nrf2 phosphorylation during *de novo* KSHV infection

KSHV infection of endothelial cells and fibroblasts induces the host's pre-existing Src, PI3-K/Akt, PKC-ζ and ERK1/2 signal pathways. This induction is important for several functions that aid in KSHV infection, including cytoskeletal rearrangements that help in virus entry and nuclear delivery, as well as viral gene expression [14]–[21]. We have also shown that ROS induction is essential for activation of FAK, Src and EphA2 receptor because pretreatment of the cells with NAC prior to KSHV infection abolished their induction [16]. Interestingly, similarly to kinase inhibitors (i.e. PP2, Wortmannin, LY294002 and Myr-PKC-ζ), ROS inhibitors such as NAC, PDTC and DPI also abolished Nrf2 phosphorylation and stability. The effect on total Nrf2 accumulation is most likely attributed to inhibition of the Keap1-Cul3 axis. The surprising effect of KSHV-induced ROS in Nrf2 phosphorylation is most likely attributed to its important role in amplifying the highly interdependent phosphorylation cascade.

Although Src, PI3-K and PKC-ζ activity was important in mediating Nrf2 activation, their inhibition affected Nrf2 behavior in distinct ways. Specifically, the Src-specific inhibitor PP2 affected Nrf2 accumulation and phosphorylation in both uninfected and infected cells, suggesting that not only is Src important for Nrf2 activity in infected cells, but also that it affects its constitutive phosphorylation levels in uninfected cells. PKC-ζ inhibition, on the other hand, did not affect total Nrf2 levels in either the infected or uninfected conditions, but it fully abolished Nrf2 phosphorylation in both cases. PI3-K inhibition did not affect Nrf2 levels or phosphorylation in uninfected cells. However, while KSHV infection of HMVEC-d cells strongly induced Nrf2 activity, infection of PI3-K-inhibited cells resulted in Nrf2 inhibition. This is a very interesting finding that can be used to affect the Nrf2 response during *de novo* infection, as modulation of a single agent, PI3-K, may be able to switch the fate of Nrf2 during *de novo* KSHV infection from induction to inhibition.

Our earlier studies have established a linear signaling pathway that starts with FAK and continues with Src, PI3-K, PKC-ζ and ERK1/2 in sequential order [6]. If the same linearity applied to the signaling cascade that mediates Nrf2 activity, one would expect that interruption of this pathway at any point during its course would have the same effect on Nrf2 stability and phosphorylation. However, given the different effects that the inhibition of Src, PI3-K and PKC-ζ kinases had on Nrf2 stability and phosphorylation (Fig. 5A–F), it is much more likely that this linear network branches off at certain sites to include additional factors important in Nrf2 activity. Interestingly, CKII, MAPK p38, JNK, etc., are kinases that may affect Nrf2 behavior, and further studies that are beyond the scope of the present one are required to decipher their role in KSHV-mediated Nrf2 activity.

### Nrf2 and host gene induction during *de novo* KSHV infection

In a previous study, we performed global host gene expression mapping at 2 and 4 hr p.i. and determined that KSHV infection of HMVEC-d cells upregulates expression of a myriad of genes involved in anti-apoptotic functions (Bcl2A1, Bcl-3), signal transduction (PKC and MAPK), cytokine signaling (IL-1β), angiogenesis (VEGF-A), and metabolism (COX-2, PFK-2) [113]. Expanding on such findings, in the current study we determined that Nrf2 was essential in upregulating the transcription of several KSHV-induced genes not previously identified, likely due to the time points selected in the previous investigations (2 and 4 hr p.i. in Naranatt *et al* (2004) vs. 8 and 24 hr p.i. in this study) [113]. Interestingly, several of the genes that KSHV induced in an Nrf2-dependent manner, such as anti-apoptosis (Bcl-2), metabolism (COX-2, G6PD, TALDO/TKT) and angiogenesis (VEGF-A and -D), fall in similar categories to those identified by Naranatt *et al* (2004) [113]. It is interesting to point out that Bcl-2 is an important anti-apoptotic factor and, like Bcl2A1, helps KSHV infection bypass apoptosis, an important barrier inherent in virus infection, as well as autophagy, a pathway well-known to antagonize KSHV infection [114].

We also identified three new KSHV-induced genes that play an important role in shunting glucose through the pentose phosphate pathway (PPP), TALDO, TKT and G6PD [49]. Such an induction could serve multiple functions, and a recent study by Mitsuishi *et al* (2012) showed that one of these functions is an increase in ribulose-5-phosphate, a crucial precursor in nucleotide synthesis [49]. This increase may provide a reservoir of nucleotides available for KSHV to utilize in i) the synthesis of host genes that are crucial in its infection or ii) the synthesis of viral genes during its early lytic burst (Fig. S4D for schematic of PPP).

Additionally, although Nrf2 was important for the transcription of several genes, they often followed different expression kinetics. For example, COX-2 and NQO1 peaked as early as 2 hr p.i., while HO1 showed no induction until 8 hr p.i. and subsided thereafter. VEGF and GCS showed Nrf2-dependence at 8 hr p.i. but seemed to be independent of Nrf2 activity at 24 hr p.i., while Bcl-2 steadily increased during the course of infection in an entirely Nrf2-dependent manner. These different patterns of transcript upregulation indicate that while Nrf2 plays an important role in the KSHV induction of these genes, assembly at the promoter of each gene is also likely affected by a cohort of gene-specific transcriptional cofactors that modulate the kinetics of each gene expression induced by Nrf2.

### Nrf2 and COX-2 link during *de novo* KSHV infection

The importance of COX-2 in KSHV biology and pathogenesis has been extensively studied [25], [84], [85], [97], [105]–[107], [109], [115]. Its induction by KSHV and the subsequent elevation in PGE2 secretion and signaling has been shown to be important for KSHV latent gene expression and KS pathogenesis. Given the importance of COX-2 in its biology, it is not surprising that KSHV utilizes Nrf2 to induce its transcriptional levels. It was interesting to observe that PGE2, an enzymatic product of COX-2, induced Nrf2 stabilization and phosphorylation through PKC-ζ activation. This is an exciting finding that establishes a self-amplifying feed-forward loop between two important agents in KSHV biology. Indeed, an initial signaling event that is initiated by virion-host receptor interaction induces Nrf2 activity, which is necessary for COX-2 upregulation and PGE2 secretion, which further induces Nrf2 activity long after the original stimulus mediated by virus binding has subsided (Fig. 14, Phase II). Such sustained Nrf2 activity could additionally help with COX-2 transcription until, and likely even after, vFLIP expression initiates. Interestingly, vFLIP was able to induce transcription of Nrf2 target genes NQO1 and HO1 when lentivirally transduced in HMVEC-d cells. The exact mechanism of vFLIP-mediated Nrf2 induction remains elusive, but it is possible that such induction is dependent on the COX-2/PGE2 axis [109].

### Nrf2 and viral gene regulation link during *de novo* KSHV infection

In long-term latent models, Ye *et al* (2011) showed that inhibiting ROS through NAC abolished TPA-induced lytic reactivation of KSHV [27]–[29]. In addition, they showed that addition of H_2_O_2_ and upregulation of endogenous ROS induced lytic reactivation of KSHV in latently infected endothelial and PEL cells, and that KSHV-induced signaling kinases were important for such activation [27]. However, the mechanisms of H_2_O_2_-induced lytic reactivation were not examined and remained unclear. Our preliminary viral gene expression profile could provide the missing link between H_2_O_2_ treatment and lytic reactivation. Our studies demonstrate that knockdown of Nrf2 results in decreased ORF50 and other lytic gene expression, especially at 8 hr p.i., and an increase in ORF73 expression, implicating Nrf2 as a positive factor for ORF50 expression (Fig. 12D–F). It could be possible that H_2_O_2_, which we showed to induce Nrf2 activity, and the kinases it induces, stabilize Nrf2 and induce its transcriptional activity on factors that upregulate lytic gene expression. It is very important to note that while these interpretations would involve Nrf2 activity as a lytic agent, such conclusions are preliminary, and current studies are underway to fully elucidate Nrf2 activity on latent and lytic gene expression in latent KSHV infection models. Moreover, the effects of Nrf2 on viral gene expression in endothelial and PEL cells may vary drastically, as developmental epigenetic modifications in these cell types are very different, leading to a completely different set of cellular cofactors present in their respective nuclei.

During *de novo* infection, virus gene expression is a dynamic process, and the role of Nrf2 on ORF73 and ORF50 expression may vary depending on time and context. Early, ORF50 (Rta) is expressed at high levels as soon as 30 min p.i., reaching a peak around 2–8 hr p.i., and subsequently declining to minimally detectable levels by 24 hr p.i. [26]. In contrast, only low levels of ORF73 (LANA-1) are detected around 2 hr p.i., but steadily increase over time, peaking around 24 hr p.i. and maintained during the observed period of 5 days p.i. [26]. Rta is shown to initiate ORF73 expression from the LTi (inducible) promoter, and subsequent LANA-1 accumulation promotes its own transcription via the LTc (constitutive) promoter while simultaneously repressing the ORF50 promoter and Rta production [5]. The low ORF50 expression in shNrf2-transduced cells and concomitant high expression of ORF73 suggest that during the early stages of KSHV infection of Nrf2-intact cells, Nrf2 could be playing a role in ORF50 induction. These events, in turn, regulate proper ORF73 expression. As LANA-1 accumulates, it could bind to Nrf2, as suggested by our PLA data (Fig. 11), possibly leading to conformational and functional changes in both proteins, modifying the positive effect that Nrf2 has on ORF50 expression. While ORF50 and other lytic gene expression in the absence of Nrf2 was significantly reduced (Fig. 12D–E and Fig. S7), their expression was still detected, likely due to the activity of other well-known ORF50 inducers such as NF-κB and ERK1/2, which in turn can induce ORF73 in an unregulated manner. Induction of very high levels of Nrf2 by H_2_O_2_ may lead to low LANA-1 and/or high ORF50 expression during *de novo* infection. Further studies are essential to dissect out the complexity of this scenario, which are beyond the scope of the current manuscript.

Overall, here we have demonstrated that KSHV induces Nrf2 during *de novo* infection of HMVEC-d cells through multiple mechanisms, and that this activation served an essential role in the induction of host and viral genes that are important in creating a microenvironment conducive to infection and establishment of latency (Fig. 14). Such knowledge, coupled to the fact that Nrf2 modulators such as Sulforaphane, tBHQ and Trigonelline are easy to administer orally, under clinical trials, and currently with no known side-effects, makes Nrf2 a very appealing target in the fight against KSHV infection.

## Materials and Methods

### Cells and tissues

HMVEC-d cells (CC-2543; Lonza Walkersville, Walkersville, MD) were cultured using endothelial basal medium (EBM2; Clonetics) supplied with growth factors (EGM2) necessary for petridish growth. Cell starvation was achieved by washing thoroughly with PBS and adding growth factor-free media for 8 hr prior to analysis. BCBL-1 cells, the source of KSHV virus used for HMVEC-d infection, were cultured in RPMI media supplied with 10% FBS and 1% penicillin-streptomycin solution as previously described [116]. Human Embryonic Kidney (HEK293T) cells were maintained in DMEM containing 1 mM pyruvate, 2 mM Glutamax, 50 u/ml penicillin, 50 mg/ml streptomycin, and 10% fetal serum (Fetalplex). Formalin-fixed, paraffin-embedded tissue samples from healthy subjects and patients with KS were obtained from the ACSR (AIDS and Cancer Specimen Resource, San Francisco, CA).

### KSHV isolation and purification

KSHV used for infection of primary HMVEC-d cells was extracted, isolated and purified from latently infected BCBL-1 cells after TPA induction as previously described [26], [116]. The quantity of KSHV DNA obtained after purification was quantified by real-time DNA PCR using KSHV ORF73 gene-specific primers as previously described [26].

### BrdU-KSHV isolation, purification and IFA detection

The pre-sterilized thymidine analogue, 5-bromo-2-deoxyuridine (BrdU labeling reagent; Ref. 00103, Invitrogen) was added to BCBL-1 cells at a 1∶100 dilution along with TPA induction and then again 24 hr later, which allowed for the metabolic labeling of the KSHV genome during lytic production. The BrdU-labeled virus was purified from the supernatant of the treated BCBL-1 cells 5 days after TPA addition as previously described [26]. For IFA detection, the usual protocol was followed and an additional step of DNA denaturing by treatment with 4N HCl for 5 minutes was performed prior to primary antibody incubation. BrdU residues were detected using mouse anti-BrdU antibody.

### EdU-KSHV isolation and purification

BCBL-1 cells were induced with neomycin and treated with 10 µM EdU dissolved in DMSO. 72 hr later, an additional dose of 10 µM EdU was supplied to the media to facilitate additional labeling. An additional 72 hr later, EdU-labeled KSHV was isolated as previously described [26]. For IFA detection, after permeabilization with 0.2% Triton X-100, the slides were incubated with Click Reaction Buffer+Cu_2_SO_4_+Alexa-Fluor-labeled Picoyl Azide for 30 minutes in the dark at RT, as per manufacturer's protocol (Life Technologies, Grand Island, NY).

### Heparin- and UV-KSHV preparation

To prepare heparin-treated virus, KSHV was incubated with heparin at a final concentration of 20 µg/ml for 1 hr at 37°C, and then used for infection of HMVEC-d cells. UV-light treatment of KSHV was performed using UV-C light for 20 minutes at a distance of 10 cm.

### Lentiviral transduction of HMVEC-d cells

Lentiviral vectors containing short hairpin RNA against Renilla luciferase (shRL), short hairpin RNA against green fluorescent protein (shGFP), short hairpin RNA against Nrf2 (shNrf2), ORF71, ORF72, ORF73, and ORFK12 were prepared using HEK293T cells as previously described [16], [117]. The supernatants containing each vector were used to transduce subconfluent HMVEC-d cells in the presence of polybrene (5 µg/ml). 24–48 hr later the cells were observed for transduction efficiency by using the transduction reporter (GFP) present in the shRL vector, and only experiments where shRL expression was present in >80% of the cells were investigated further. Knockdown was also verified using real-time RT-PCR analysis or WB for Nrf2.

### Antibodies and reagents

Antibodies against tNrf2, NQO1, pPKC-ζ and pPKC-ζ, and Tyr-216 GSK-3β were obtained from Santa Cruz Biotechnology Inc., Santa Cruz, CA, whereas antibodies for tGSK-3β, Ser-9 GSK-3β, pNF-κB (Ser-536), ERK1/2 and pERK1/2 were obtained from Cell Signaling Technologies, Danvers, MA. pNrf2, Keap1 and p62 antibodies were obtained from Abcam, Boston, MA. Antibodies against β-actin, β-tubulin and TBP were obtained from Sigma-Aldrich, St. Louis, MO. The Ub-K48 antibody was from Millipore, Billerica, MA, and the mouse anti-BrdU antibody was from Life Technologies. ORF50 antibody was obtained from ABBIOTEC, San Diego, CA. HRP-linked anti-mouse and anti-rabbit antibodies used for chemiluminescent detection of Western blot bands were from KPL Inc, Gaithersburg, MD. 49,6-diamidino-2- phenylindole (DAPI), anti-rabbit and anti-mouse Alexa-Fluor 594 or 488 secondary antibodies were from Molecular Probes, Carlsbad, CA. Chemical inhibitors PP2, LY294002, Wortmannin, U73122, Myr-PKC-ζ, U0126, PGE2, and Bay-11-7082 were from Cayman Chemicals, Ann Arbor, MI. Nuclear Extract and TransAM Nrf2 Kits were from Active Motif, Carlsbad, CA. Nuclei EZ Prep Nuclei Isolation Kit was from Sigma-Aldrich. The Human VEGF Quantikine ELISA Kit was from R&D Systems Minneapolis, MN. Heparin, H_2_O_2_, N-acetylcysteine (NAC), pyrrolidine dithiocarbamate (PDTC) and Trigonelline were obtained from Sigma-Aldrich. The CM-H_2_CDFDA ROS-measuring dye was obtained from Life Technologies.

### Western blotting

At the end of treatment, cells were suspended in RIPA Lysis Buffer (25 mM Tris-HCl, pH = 7.6, 150 mM NaCl, 1% NP-40, 0.1% SDS, and protease/phosphatase inhibitor cocktails). The lysates were sonicated to shear the DNA remnants and centrifuged at max-speed for 20 minutes at 4°C to rid the insoluble fractions. Protein concentration was assessed using BCA protein assay reagent (Pierce, Rockford, IL). Equal amounts of protein were separated by SDS-PAGE, transferred to a nitrocellulose membrane and probed using protein-specific primary and species-specific, HRP-conjugated, secondary antibodies. Detection was assessed by chemiluminescence assays (Pierce) according to manufacturer's protocol. The bands were digitalized using an Alpha-Imager System (Alpha Innotech Corporation, San Leonardo, CA) and quantified by ImageJ Software.

### Isolation of nuclear and cytoplasmic fractions for western blot analysis

HMVEC-d cells were infected with KSHV (20 DNA copies/cell) at 37°C for different time points, and the nuclear and cytoplasmic proteins were fractioned per the manufacturer's protocol (Nuclear Isolation Kit, Active Motif). Briefly, cells were trypsinized, washed with PBS supplied with phosphatase inhibitor, lysed with 1× Hypotonic Buffer to disrupt the plasma membrane and collect the cytoplasmic fraction after pelleting the nuclei by centrifugation at 500×g for 2 minutes. The nuclear pellet was washed 2 additional times with 1× Hypotonic Buffer to remove any loosely bound cytoplasmic contaminants prior to resuspension in complete lysis buffer.

### Co-immunoprecipitation

Cells were lysed either with denaturing RIPA buffer or non-denaturing buffer (NETM – 100 mM NaCl, 20 mM Tris-HCl, pH = 8.0, 0.5 mM EDTA and 0.5% NP-40) and 200 µg of protein was incubated with 1–2 µg of primary antibody, along with Protein G-sepharose beads. After overnight incubation at 4°C, the beads were pelleted by centrifugation, washed 3 times with the original lysis buffer, and the protein-bead complex was disrupted by resuspending them in SDS-Loading buffer solution and heating to 95°C for 10 minutes prior to SDS-PAGE gel loading and Western blot analysis.

### Measurement of KSHV entry by real-time DNA PCR of ORF73

Starved HMVEC-d cells were infected with KSHV (20 DNA copies/cell) for 30 minutes, washed with trypsin-EDTA to remove non-internalized virus, and total genomic and viral DNA was isolated using a DNeasy Blood and Tissue Kit (Qiagen) according to manufacturer's protocol. ORF73 copy number was quantified by real-time DNA PCR amplification from equal amounts of DNA from each condition as previously described [26]. The ORF73 gene cloned in the pGEM-T vector (Promega) was used to create a standard curve to determine the absolute DNA copy number of unknown conditions.

### Measurement of KSHV nuclear delivery by real-time DNA PCR of ORF73

Starved HMVEC-d cells were infected with KSHV (20 DNA copies/cell) for 2 hr, washed with trypsin-EDTA, and subjected to nuclear isolation using a Nuclei EZ Prep Nuclei Isolation Kit (Sigma-Aldrich) as previously described [26]. Briefly, cells were lysed with mild lysis buffer and the nuclei were pelleted by centrifugation at 500×g for 5 minutes. The cytoplasmic contaminants that were loosely attached to the nuclei were removed by repeated washing with the mild lysis buffer. The DNA was isolated using the DNeasy Blood & Tissue Kit (Qiagen). Equal amounts of DNA were quantified with ORF73-specific primers by real-time DNA PCR. The absolute copy number was determined using an ORF73 standard curve as described in the entry experiment.

### Measurement of KSHV gene expression by one-step real-time PCR

To assess the gene expression of the two major viral genes, ORF50 and ORF73, we extracted total RNA from KSHV-infected cells (50 DNA copies/cell) using RNeasy Mini Kit (Qiagen) spin columns. Equal amounts of RNA (2–4 µg), as determined by NanoDrop quantification, were subjected to one-step real-time RT-PCR analysis using ORF50- or ORF73-specific primers and Taqman probes (EZ RT-PCR core reagents, Applied Biosystems) as previously described [26]. The absolute copy number of each mRNA was assessed using ORF50 and ORF73-specific RNA standard curves obtained from *in vitro*-derived transcripts as previously described [26].

### Measurement of host gene expression by real-time RT-PCR

To assess changes in host gene expression, total RNA from infected cells was isolated using the RNeasy Mini Kit (Qiagen), and a cDNA library was created using a High-Capacity cDNA Reverse Transcription Kit (Life Technologies). The cycle threshold (Ct) values for each gene were determined using gene-specific primers and Sybr Green Probe-based real-time RT-PCR. ΔCt values relative to β-tubulin were assessed for each condition. Gene expression in the uninfected/untreated conditions was arbitrarily set to 1, and fold-induction was based on the ΔCt differential relative to this condition.

### Immunofluorescence microscopy

HMVEC-d cells grown in 8 chamber glass slides (Nalge Nunc International) were fixed with 4% paraformaldehyde for 15 minutes at room temperature and then permeabilized with 0.2% Triton X-100 for 5 minutes. The cells were then blocked with Image-iT FX signaling enhancer (Life Technologies) for 15 minutes prior to incubation with specific primary antibodies and fluorescent Alexa-Fluor-conjugated secondary antibodies (Alexa-Fluor) and mounting with DAPI for nuclear staining. Regular imaging was performed with Nikon imaging systems and figure analysis and deconvolution of the images was performed using the Metamorph software. Confocal imaging was performed using the Olympus FV10i microscope, and image analysis was performed using Fluoview1000 (Olympus) software.

### VEGF ELISA

To determine the level of VEGF secretion in infected cells, serum-starved HMVEC-d cells' supernatant was collected at various time points, spun at 1,000×rpm to remove any cellular debris, and frozen at −80°C until further use. Levels of the protein were assessed using a Human VEGF Quantikine ELISA Kit per manufacturer's protocol (R & D Systems).

### Nrf2 ELISA

To assess the DNA-binding activity of nuclear Nrf2 we first isolated the nuclear protein fraction using the Nuclear Extraction Kit (Active Motif), and performed Western blot analysis to assess the quality of the fraction by using the TATA Binding Protein (TBP) as a nuclear positive control, β-tubulin as a negative control, and actin as a loading control. The DNA-binding activity of Nrf2 was assessed using TransAM Nrf2 Kit as per manufacturer's instructions (Active Motif). Briefly, equal amounts of nuclear protein (15 µg/condition), as determined by BCA assay, were loaded in each well containing the Nrf2-binding oligo probes (TGAnnnnGC) for 1 hr at room temperature, washed, and sandwiched with an HRP-conjugated anti-Nrf2 antibody for an additional hour prior to chemiluminescent assessment. Thirty µg of MCF-7 cell line nuclear extract was used as a positive control for the assay.

### ROS measurement

Starved HMVEC-d cells cultured in a 48-well plate were infected for various time points prior to assessing ROS levels as previously described [16]. Briefly, EBM2 medium containing 10 µM of the ROS-detecting dye 5-(and-6)-cholormethyl-2′,7′-dichlorohydrofluorescein diacetate, acetyl ester (CM-H_2_DCFDA [C6827]); Invitrogen) was added to untransduced, shRL and shNrf2 cells for 30 minutes prior to infection with KSHV (40 DNA copies/cell). Fluorescence measurement and calculations were performed per manufacturer's protocol as previously described [16].

### *In situ* proximity ligation assay (PLA) microscopy

PLA (DuoLink, Sigma-Aldrich) was performed using the DuoLink PLA Kit to detect protein–protein interactions using fluorescence microscopy as per manufacturer's protocol. Briefly, HMVEC-d cells were cultured and infected with KSHV (20 DNA copies/cell) for 24 hr in 8 chamber microscopic slides, fixed with 4% paraformaldehyde for 15 minutes at room temperature, permeabilized with 0.2% Triton X-100 and blocked with DuoLink blocking buffer for 30 minutes at 37°C. Cells were then incubated with primary antibodies against LANA-1 (mouse monoclonal) and pNrf2 (rabbit) diluted in DuoLink antibody diluents for 1 hr, washed and then further incubated for another hour at 37°C with species-specific PLA probes under hybridization conditions and in the presence of 2 additional oligonucleotides to facilitate the hybridization only in close proximity (<16 nm). A ligase was then added to join the two hybridized oligonucleotides to form a closed circle and initiate a rolling-circle amplification using the ligated circle as a template after adding an amplification solution to generate a concatemeric product extending from the oligonucleotide arm of the PLA probe. Lastly, a detection solution consisting of fluorescently labeled oligonucleotides was added, and the labeled oligonucleotides were hybridized to the concatemeric products. The signal was detected as distinct fluorescent dots in the Texas red channel and analyzed by fluorescence microscopy. Negative controls consisted of samples treated as described but with only secondary antibodies.

## Supporting Information

Figure S1 **The Nrf2-Keap1-Cul3 interaction model.** **A**) In an unstressed cell, (i) the constitutively translated Nrf2 protein is maintained under control by the Keap1-Cul3 ubiquitination machinery. The two Kelch domains of a Keap1 homodimer bind to the Neh2 domain of one Nrf2 molecule, whereas the BTB domains of Keap1 bind to Cul3. This Nrf2-Keap1-Cul3 complex cycles between two conformations, (ii) closed, during which time the Cul3 E3-ubiquitin ligase mediates Nrf2 ubiquitination, and (iii) open, during which time the ubiquitinated Nrf2 gets degraded by the 26S proteasome. As a result, the Keap1-Cul3 complex is recycled and ready to mediate ubiquitination and degradation of another, newly translated, Nrf2 molecule, maintaining low basal Nrf2 levels. **B**) During times of oxidative stress, alteration in the Keap1 cysteine redox state induces conformational changes that shift the closed-to-open cycling of the Nrf2-Keap1-Cul3 complex towards the closed conformation irrespective of the ubiquitination status of Nrf2 (ii and iii). This impairs the ability of the proteasome to access ubiquitinated Nrf2, which in turn, keeps the Keap1-Cul3 ubiquitination machinery hostage. No recycling of this complex results in its quick saturation, and as a consequence, newly synthesized Nrf2 accumulates in the cell free of degradation by the proteasome (i).
(TIF)

Figure S2 **Signaling, ROS and Nrf2 stability.** **A**) HMVEC-cells infected with KSHV (20 DNA copies/cell) were immunoblotted with the well-known KSHV-induced marker pPKC-ζ. For loading control, refer to Fig. 2A. **B**) HMVEC-d cells were pretreated with DPI (50 µM) for 2 hr prior to infection with KSHV (20 DNA copies/cell) for an additional 2 hr before immunoblotting with pNF-κB (Ser-536) and NF-κB. **C**) *Left:* HMVEC-d cells were starved and treated with NAC (10 mM) or PDTC (100 µM) for 2 hr prior to infection for an additional 2 hr. The cells were then placed in growth factor-supplied media supplemented with NAC (2.5 mM) or PDTC (25 µM) overnight and starved for an additional 8 hr before immunoblot analysis. *Right:* Starved HMVEC-d cells were first infected with KSHV for 16 hr in the absence of any inhibitors, then starved in the presence of NAC (10 mM) or PDTC (100 µM) for 8 hr prior to immunoblot analysis. **D**) Starved HMVEC-d cells infected with KSHV in the absence (left panels) or presence of 10 mM NAC (middle panel) or 100 µM PDTC (right panel) analyzed by immunofluorescence assay and stained with anti-pNrf2 primary antibody and anti-rabbit Alexa-Fluor 488 secondary antibody (green). Yellow square = enlarged area; blue staining = DAPI; pNrf2 = phosphorylated/active form of Nrf2; NAC = *N*-*A*cetylcysteine; PDTC = Pyrrolidine Dithiocarbamate.
(TIF)

Figure S3 **The role of KSHV binding to cell-surface receptors in Nrf2 induction.** IFA of pNrf2 localization and levels in HMVEC-d cells infected with functional KSHV, heparin-treated KSHV. Starved cells were infected with each virus for the indicated time points, and the slides were stained with rabbit anti-pNrf2 primary antibody and goat anti-rabbit (Alexa-Fluor 488 – green) secondary antibody. Yellow square = enlarged area; red arrow = nuclear localization; white arrow = cytoplasmic localization; blue staining = DAPI.
(TIF)

Figure S4 **Lentiviral shNrf2 knockdown efficiency.** **A**) HMVEC-d cells were transduced with lentiviral vectors containing shRL or shNrf2 for 72 hr and then infected with KSHV for 2 hr. pNrf2 and tNrf2 were used to detect the efficiency, while ERK1/2 and β-actin were used to assess the specificity of the knockdown. **B–C**) Real-time RT-PCR analysis of Nrf2 target genes involved in **B**) apoptosis (Bcl-2), and **C**) the pentose phosphate pathway (G6PD, TALDO and TKT). β-tubulin was used as an endogenous control, the uninfected condition (U.I.) was arbitrarily set to 1, and the bars indicate mean fold induction ± SD for 4 independent experiments. * = p<0.05. (G6PD = Glucose-6-Phosphate Dehydrogenase; TKT = Transketolase; TALDO = Transaldolase). **D**) Diagram depicting the roles of G6PD, TKT and TALDO in the PPP and nucleic acid synthesis.
(TIF)

Figure S5 **Role of Nrf2 in NF-κB induction by KSHV.** HMVEC-d cells were initially transduced with shRL/shNrf2-containing vectors for 72 hr prior to infection with KSHV (20 DNA copies/cell) before immunoblotting with pNF-κB (Ser-536) and total NF-κB.
(TIF)

Figure S6 **Verification of lentiviral expression of latent KSHV genes.** **A**) Starved HMVEC-d cells were infected with KSHV or UV-treated KSHV for 2 and 24 hr prior to immunofluorescence analysis. The slides were stained with rabbit anti-pNrf2 primary antibody and goat anti-rabbit (Alexa-Fluor 488 – green) secondary antibody. Yellow square = enlarged area; red arrow = nuclear localization; white arrow = cytoplasmic localization; blue staining = DAPI. **B**) HMVEC-d cells were transduced with lentiviral vectors containing ORF71/vFLIP, ORF72/vCyclin, ORF73/LANA-1, ORFK12/Kaposin and pSIN (empty vector) for 72 hr prior to confirmation with real-time RT-PCR using primers specific for each gene.
(TIF)

Figure S7 **Nrf2 modulation and KSHV entry.** (**A and B**) Real-time PCR of Nrf2 mRNA to determine the efficiency of lentiviral knockdown for the (**A**) entry and (**B**) ROS experiments in figures 12A and 12B. shRL/shGFP were arbitrarily set to 1 and bars indicate mean ± SD for 3 replicates. * = p<0.05. **C**) Real-time PCR analysis using NQO1 and GCS-specific primers on RNA extracted from cells infected with KSHV in the absence (black and red bars) or presence of 0.5 µM Trigonelline (blue bars). **D**) Western blot analysis of pNrf2 and tNrf2 in HMVEC-d cells treated with the Nrf2 inducers tBHQ and Sulforaphane for the indicated doses and times. β-actin was used as a loading control. **E**) Starved HMVEC-d cells were treated with the Nrf2 inhibitor, Trigonelline (0.5 µM), Nrf2 inducers Sulforaphane (100 µM) and tBHQ (10 µM) and NF-κB inhibitor, Bay-11-7082 (1 µM) for 4 hr prior to performing a KSHV entry assay. Briefly, cells were infected with KSHV (20 DNA copies/cell) for 30 min at 37°C, washed 3 times with PBS, trypsinized to remove non-internalized virus, and the DNA was isolated using a DNeasy Blood & Tissue Kit (Qiagen). DNA real-time PCR was performed with ORF73 gene-specific primers and the absolute KSHV copy number was calculated from a standard curve obtained by real-time PCR of standards with known concentrations of ORF73. **F–G**) Starved HMVEC-d cells in a 48-well plate were incubated with 10 µM CM-H_2_DCFDA (ROS-measuring dye) for 30 min at 37°C, and then infected with KSHV (40 DNA copies/well). ROS levels were assessed early (F) or late (G) by using a 488/20 excitation and 528/20 emission filter pair and a PMT sensitivity setting of 55. Values indicate mean ± SD for 3 independent replicates.
(TIF)

Figure S8 **Nrf2 modulation and KSHV gene expression.** **A**) Real-time PCR of Nrf2 mRNA to determine the efficiency of lentiviral knockdown for the nuclear delivery experiment. ShRL was arbitrarily set to 1 and the bars indicate mean ± SD for 3 replicates. * = p<0.05 when compared to shRL. **B**) Starved HMVEC-d cells were treated with the Nrf2 inhibitor Trigonelline (0.5 µM), Nrf2 inducers Sulforaphane (100 µM) and tBHQ (10 µM) and NF-κB inhibitor Bay-11-7082 (1 µM) for 4 hr prior to infection with KSHV (20 DNA copies/cell) for 2 hr. The nucleus-associated DNA was obtained using a DNeasy Blood & Tissue Kit (Qiagen) on nuclei isolated with Nuclei EZ Prep Nuclei Isolation Kit (Sigma-Aldrich) following the manufacturer's protocol. DNA real-time PCR was performed using ORF73 gene-specific primers to determine the levels of viral DNA. The absolute copy number was calculated from a standard curve obtained by real-time PCR of known standards with known concentrations of ORF73. Bars indicate mean ± SD for 3 independent replicates. **C–D**) Starved HMVEC-d cells were infected with KSHV (20 DNA copies/cell) prior to RNA isolation. One-step real-time PCR was performed on the viral genes **C**) ORF73 and **D**) ORF50. The absolute copy number was calculated from a standard curve obtained by real-time PCR of RNA standards of ORF73 or ORF50 with known concentrations. Bars indicate mean copy number ± SD of 3 independent replicates. **E–F**) Real-time PCR using specific primers for Nrf2 and NQO1 on RNA isolates of cells described in experiments under Figures 9E and F. Nrf2 PCR was performed to verify the knockdown in shNrf2 cells and NQO1 to determine the induction of Nrf2 in shRL cells.
(TIF)

Figure S9 **KSHV early lytic burst gene expression during Nrf2 knockdown.** HMVEC-cells were initially transduced with shRL/shNrf2-containing lentiviral vectors for 72 hr prior to infection with KSHV (50 DNA copies/cell). The levels of various lytic genes such as ORF50, K8, K5 and vIRF2 were used using gene-specific primers by real-time RT-PCR.
(TIF)

Figure S10 **GSK-3β and its role in Nrf2 stability during** ***de novo*** **KSHV infection.** HMVEC-d cells were infected with KSHV (20 DNA copies/cell) and immunoblotted for p62, pGSK-3β (Y-216), pGSK-3β (S-9) and tGSK-3β. Fold induction normalized to β-tubulin and relative to the uninfected (U.I.) condition (arbitrarily set to 1) are indicated.
(TIF)
