# Supplementary figures and images for: Kaposi's Sarcoma-Associated Herpesvirus Induces Nrf2 during De Novo Infection of Endothelial Cells to Create a Microenvironment Conducive to Infection
Source: PLoS Pathog. 2014 Oct 23;10(10):e1004460. doi: 10.1371/journal.ppat.1004460 (PMC4207826; doi:10.1371/journal.ppat.1004460)

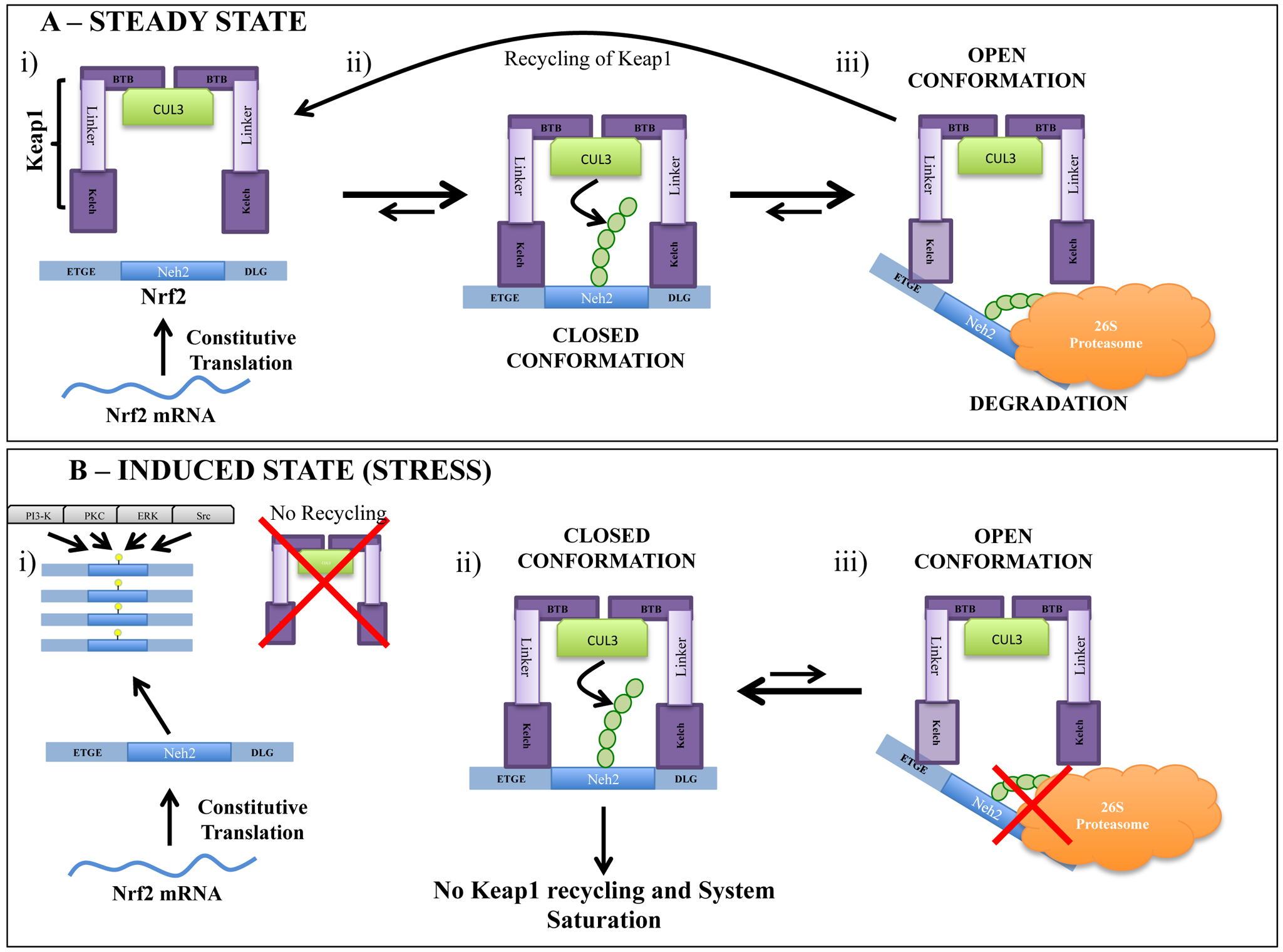

Supplement: Figure S1 — The Nrf2-Keap1-Cul3 interaction model. A) In an unstressed cell, (i) the constitutively translated Nrf2 protein is maintained under control by the Keap1-Cul3 ubiquitination machinery. The two Kelch domains of a Keap1 homodimer bind to the Neh2 domain of one Nrf2 molecule, whereas the BTB domains of Keap1 bind to Cul3. This Nrf2-Keap1-Cul3 complex cycles between two conformations, (ii) closed, during which time the Cul3 E3-ubiquitin ligase mediates Nrf2 ubiquitination, and (iii) open, during which time the ubiquitinated Nrf2 gets degraded by the 26S proteasome. As a result, the Keap1-Cul3 complex is recycled and ready to mediate ubiquitination and degradation of another, newly translated, Nrf2 molecule, maintaining low basal Nrf2 levels. B) During times of oxidative stress, alteration in the Keap1 cysteine redox state induces conformational changes that shift the closed-to-open cycling of the Nrf2-Keap1-Cul3 complex towards the closed conformation irrespective of the ubiquitination status of Nrf2 (ii and iii). This impairs the ability of the proteasome to access ubiquitinated Nrf2, which in turn, keeps the Keap1-Cul3 ubiquitination machinery hostage. No recycling of this complex results in its quick saturation, and as a consequence, newly synthesized Nrf2 accumulates in the cell free of degradation by the proteasome (i). (TIF) [file ppat.1004460.s001.tif]

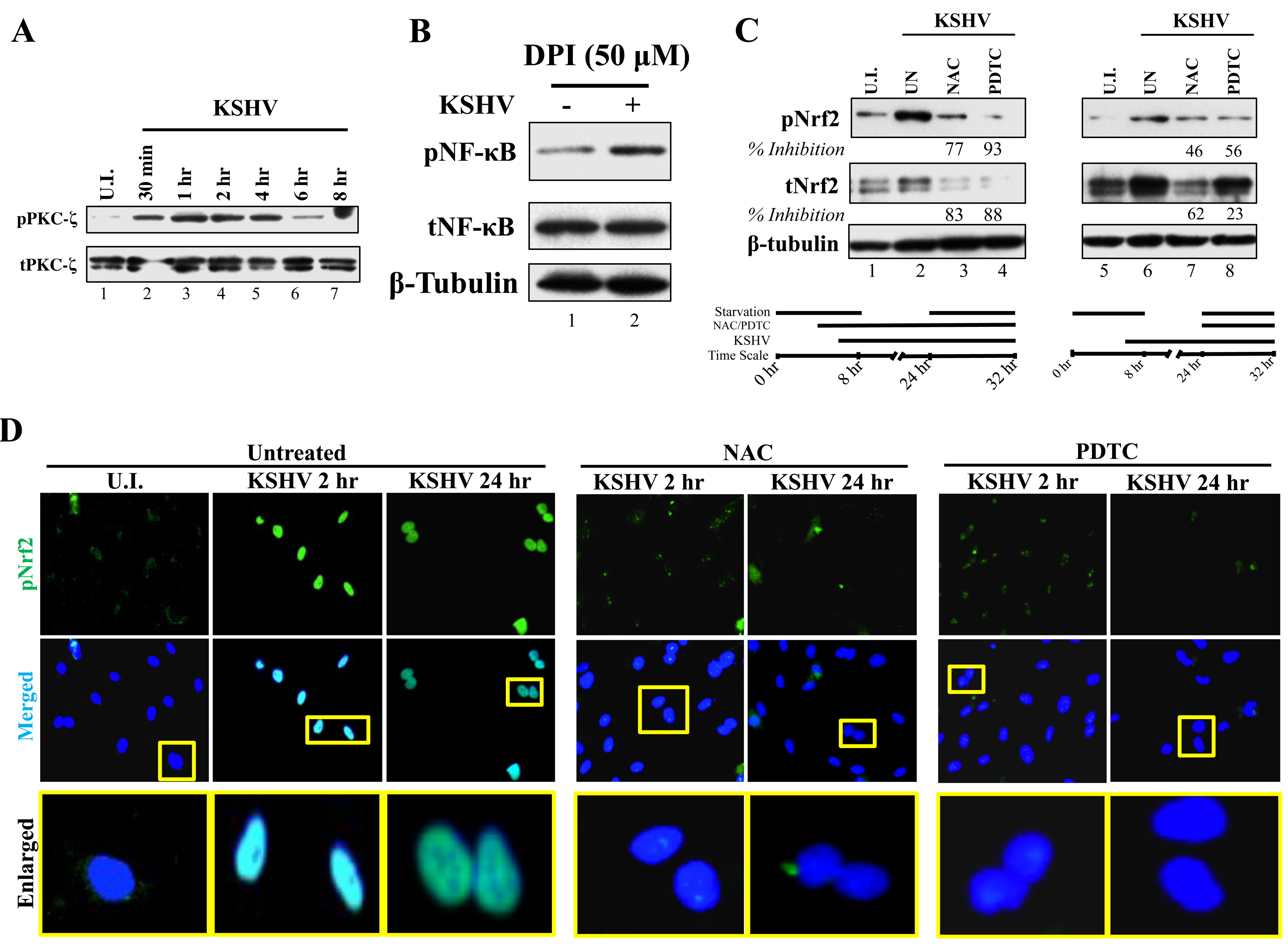

Supplement: Figure S2 — Signaling, ROS and Nrf2 stability. A) HMVEC-cells infected with KSHV (20 DNA copies/cell) were immunoblotted with the well-known KSHV-induced marker pPKC-ζ. For loading control, refer to Fig. 2A. B) HMVEC-d cells were pretreated with DPI (50 µM) for 2 hr prior to infection with KSHV (20 DNA copies/cell) for an additional 2 hr before immunoblotting with pNF-κB (Ser-536) and NF-κB. C) Left: HMVEC-d cells were starved and treated with NAC (10 mM) or PDTC (100 µM) for 2 hr prior to infection for an additional 2 hr. The cells were then placed in growth factor-supplied media supplemented with NAC (2.5 mM) or PDTC (25 µM) overnight and starved for an additional 8 hr before immunoblot analysis. Right: Starved HMVEC-d cells were first infected with KSHV for 16 hr in the absence of any inhibitors, then starved in the presence of NAC (10 mM) or PDTC (100 µM) for 8 hr prior to immunoblot analysis. D) Starved HMVEC-d cells infected with KSHV in the absence (left panels) or presence of 10 mM NAC (middle panel) or 100 µM PDTC (right panel) analyzed by immunofluorescence assay and stained with anti-pNrf2 primary antibody and anti-rabbit Alexa-Fluor 488 secondary antibody (green). Yellow square = enlarged area; blue staining = DAPI; pNrf2 = phosphorylated/active form of Nrf2; NAC = N-Acetylcysteine; PDTC = Pyrrolidine Dithiocarbamate. (TIF) [file ppat.1004460.s002.tif]

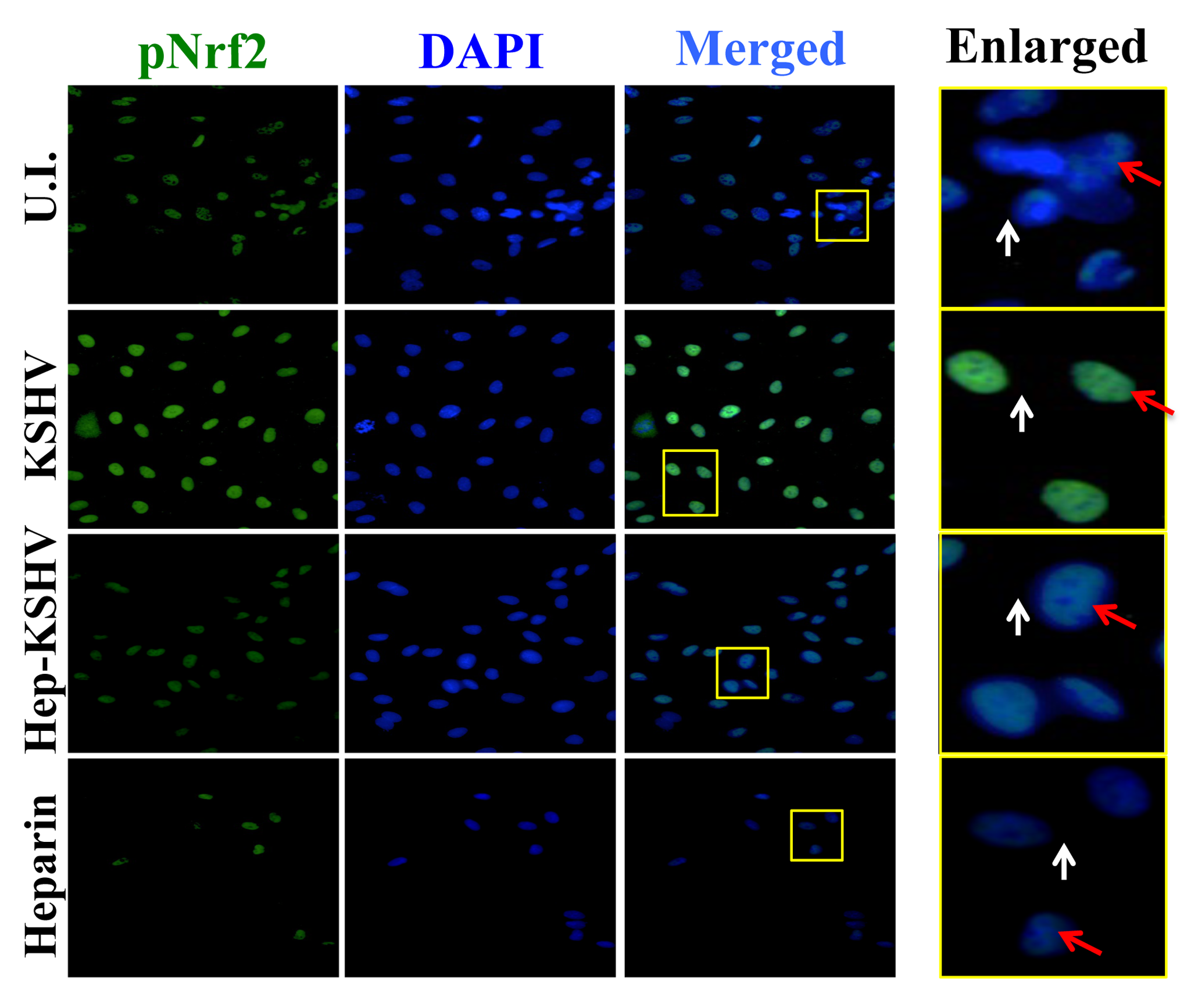

Supplement: Figure S3 — The role of KSHV binding to cell-surface receptors in Nrf2 induction. IFA of pNrf2 localization and levels in HMVEC-d cells infected with functional KSHV, heparin-treated KSHV. Starved cells were infected with each virus for the indicated time points, and the slides were stained with rabbit anti-pNrf2 primary antibody and goat anti-rabbit (Alexa-Fluor 488 – green) secondary antibody. Yellow square = enlarged area; red arrow = nuclear localization; white arrow = cytoplasmic localization; blue staining = DAPI. (TIF) [file ppat.1004460.s003.tif]

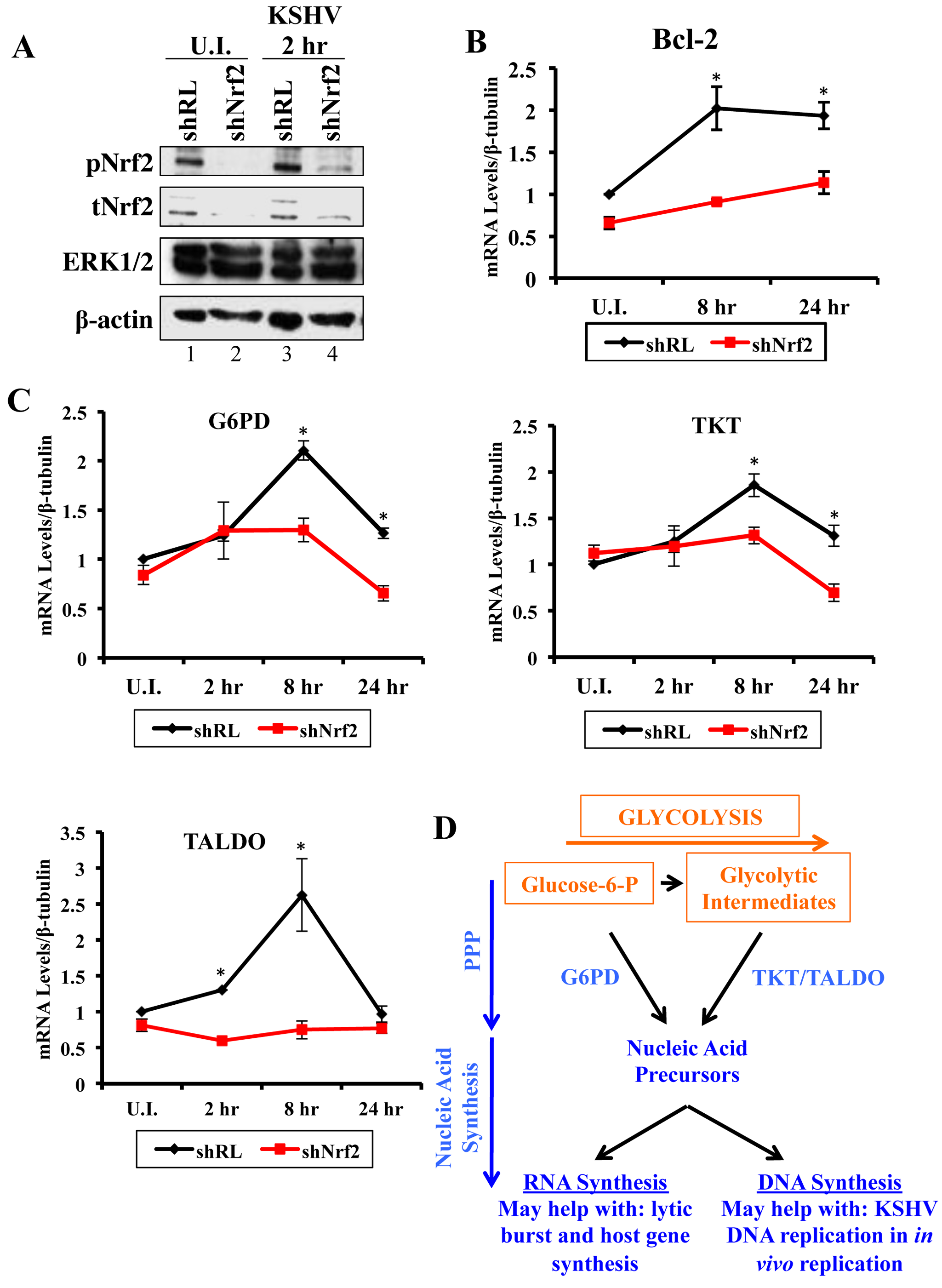

Supplement: Figure S4 — Lentiviral shNrf2 knockdown efficiency. A) HMVEC-d cells were transduced with lentiviral vectors containing shRL or shNrf2 for 72 hr and then infected with KSHV for 2 hr. pNrf2 and tNrf2 were used to detect the efficiency, while ERK1/2 and β-actin were used to assess the specificity of the knockdown. B–C) Real-time RT-PCR analysis of Nrf2 target genes involved in B) apoptosis (Bcl-2), and C) the pentose phosphate pathway (G6PD, TALDO and TKT). β-tubulin was used as an endogenous control, the uninfected condition (U.I.) was arbitrarily set to 1, and the bars indicate mean fold induction ± SD for 4 independent experiments. * = p<0.05. (G6PD = Glucose-6-Phosphate Dehydrogenase; TKT = Transketolase; TALDO = Transaldolase). D) Diagram depicting the roles of G6PD, TKT and TALDO in the PPP and nucleic acid synthesis. (TIF) [file ppat.1004460.s004.tif]

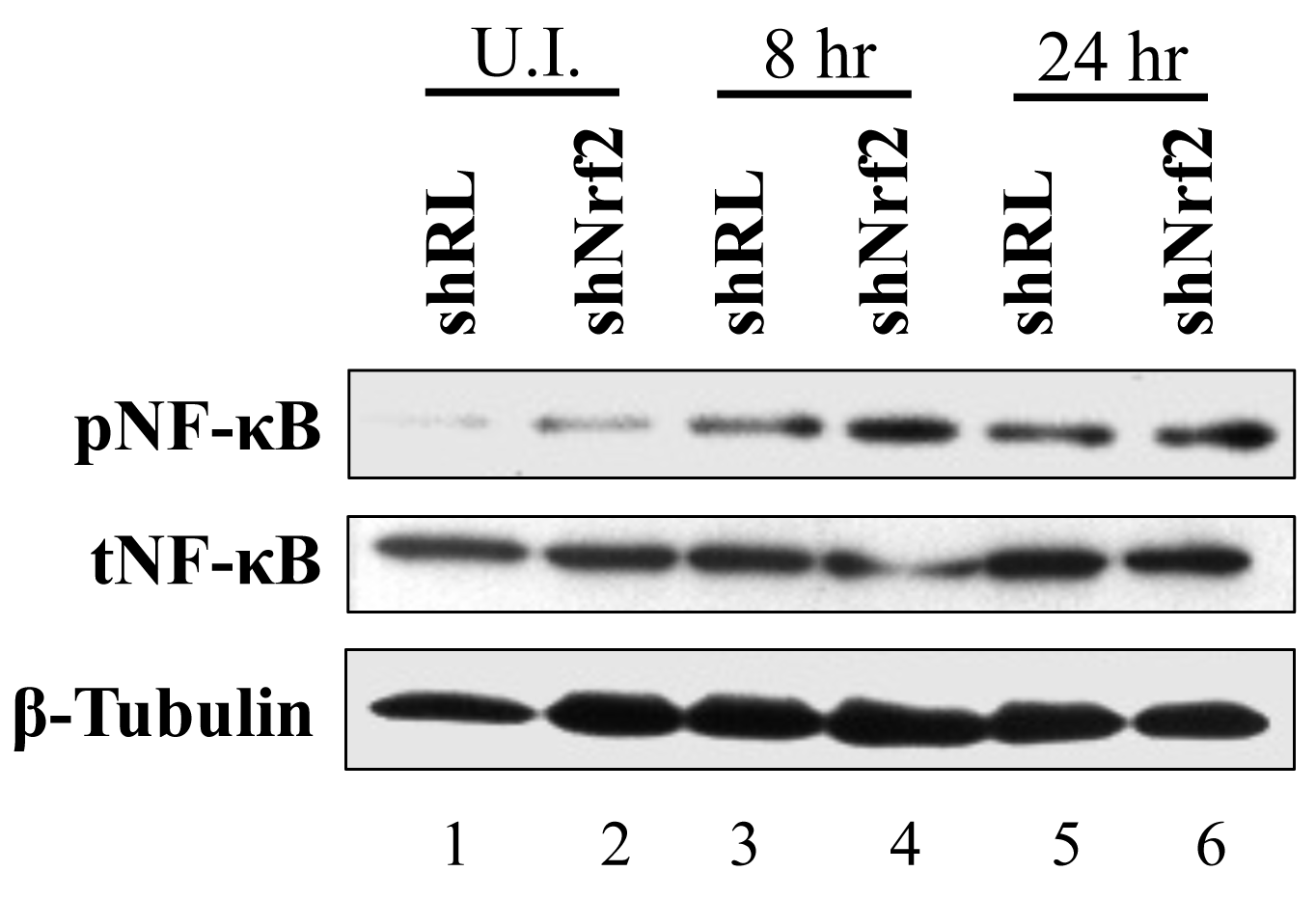

Supplement: Figure S5 — Role of Nrf2 in NF-κB induction by KSHV. HMVEC-d cells were initially transduced with shRL/shNrf2-containing vectors for 72 hr prior to infection with KSHV (20 DNA copies/cell) before immunoblotting with pNF-κB (Ser-536) and total NF-κB. (TIF) [file ppat.1004460.s005.tif]

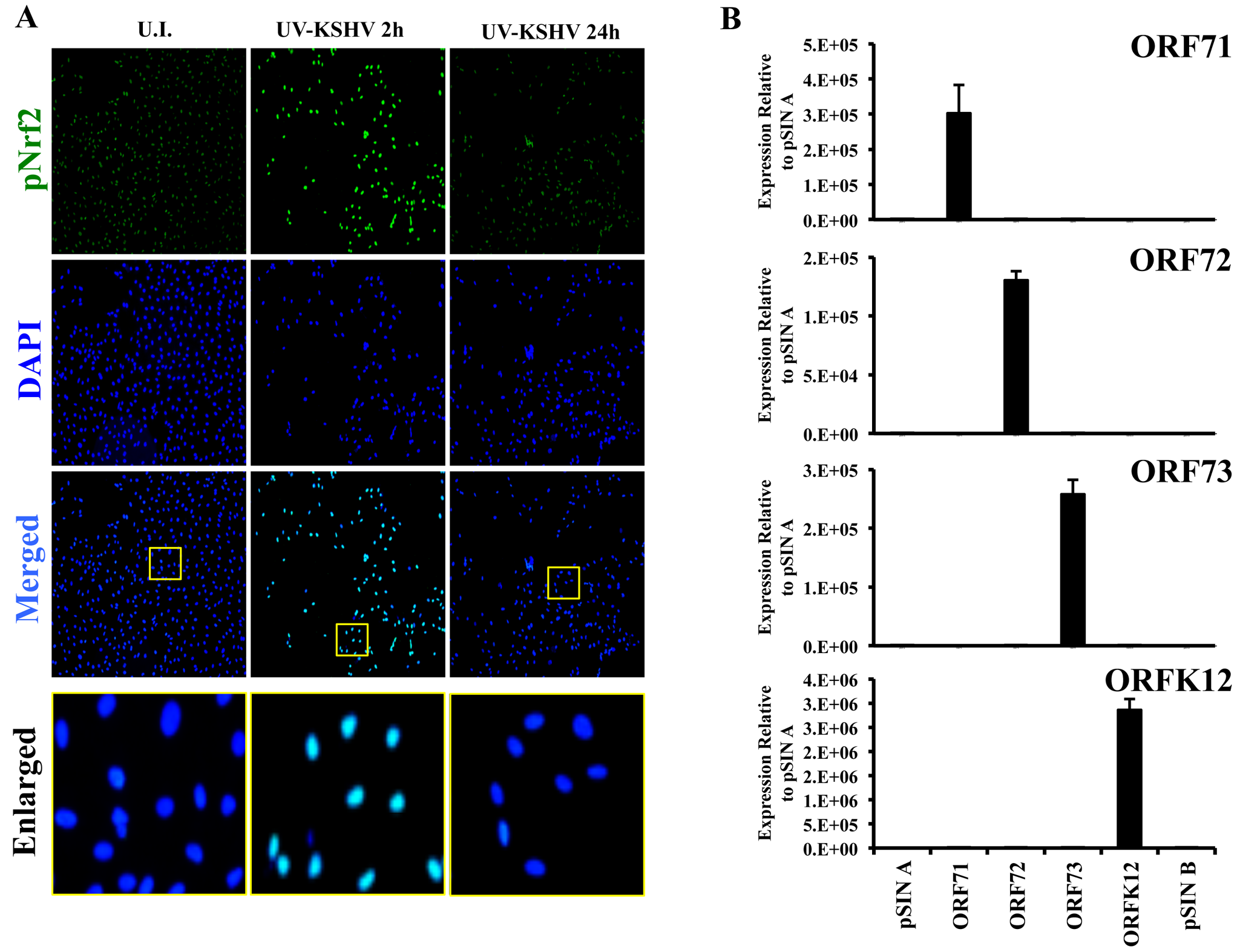

Supplement: Figure S6 — Verification of lentiviral expression of latent KSHV genes. A) Starved HMVEC-d cells were infected with KSHV or UV-treated KSHV for 2 and 24 hr prior to immunofluorescence analysis. The slides were stained with rabbit anti-pNrf2 primary antibody and goat anti-rabbit (Alexa-Fluor 488 – green) secondary antibody. Yellow square = enlarged area; red arrow = nuclear localization; white arrow = cytoplasmic localization; blue staining = DAPI. B) HMVEC-d cells were transduced with lentiviral vectors containing ORF71/vFLIP, ORF72/vCyclin, ORF73/LANA-1, ORFK12/Kaposin and pSIN (empty vector) for 72 hr prior to confirmation with real-time RT-PCR using primers specific for each gene. (TIF) [file ppat.1004460.s006.tif]

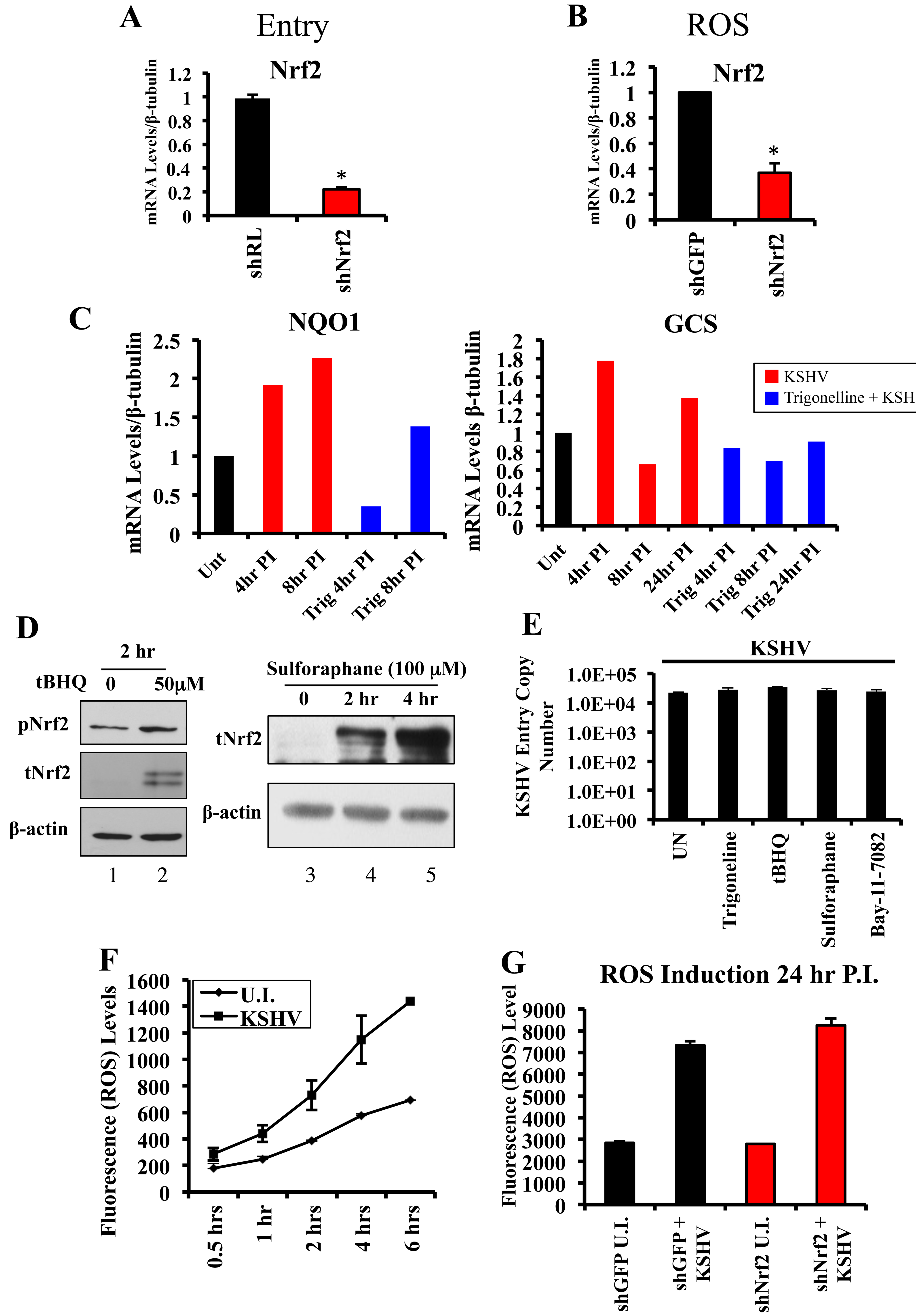

Supplement: Figure S7 — Nrf2 modulation and KSHV entry. (A and B) Real-time PCR of Nrf2 mRNA to determine the efficiency of lentiviral knockdown for the (A) entry and (B) ROS experiments in figures 12A and 12B. shRL/shGFP were arbitrarily set to 1 and bars indicate mean ± SD for 3 replicates. * = p<0.05. C) Real-time PCR analysis using NQO1 and GCS-specific primers on RNA extracted from cells infected with KSHV in the absence (black and red bars) or presence of 0.5 µM Trigonelline (blue bars). D) Western blot analysis of pNrf2 and tNrf2 in HMVEC-d cells treated with the Nrf2 inducers tBHQ and Sulforaphane for the indicated doses and times. β-actin was used as a loading control. E) Starved HMVEC-d cells were treated with the Nrf2 inhibitor, Trigonelline (0.5 µM), Nrf2 inducers Sulforaphane (100 µM) and tBHQ (10 µM) and NF-κB inhibitor, Bay-11-7082 (1 µM) for 4 hr prior to performing a KSHV entry assay. Briefly, cells were infected with KSHV (20 DNA copies/cell) for 30 min at 37°C, washed 3 times with PBS, trypsinized to remove non-internalized virus, and the DNA was isolated using a DNeasy Blood & Tissue Kit (Qiagen). DNA real-time PCR was performed with ORF73 gene-specific primers and the absolute KSHV copy number was calculated from a standard curve obtained by real-time PCR of standards with known concentrations of ORF73. F–G) Starved HMVEC-d cells in a 48-well plate were incubated with 10 µM CM-H2DCFDA (ROS-measuring dye) for 30 min at 37°C, and then infected with KSHV (40 DNA copies/well). ROS levels were assessed early (F) or late (G) by using a 488/20 excitation and 528/20 emission filter pair and a PMT sensitivity setting of 55. Values indicate mean ± SD for 3 independent replicates. (TIF) [file ppat.1004460.s007.tif]

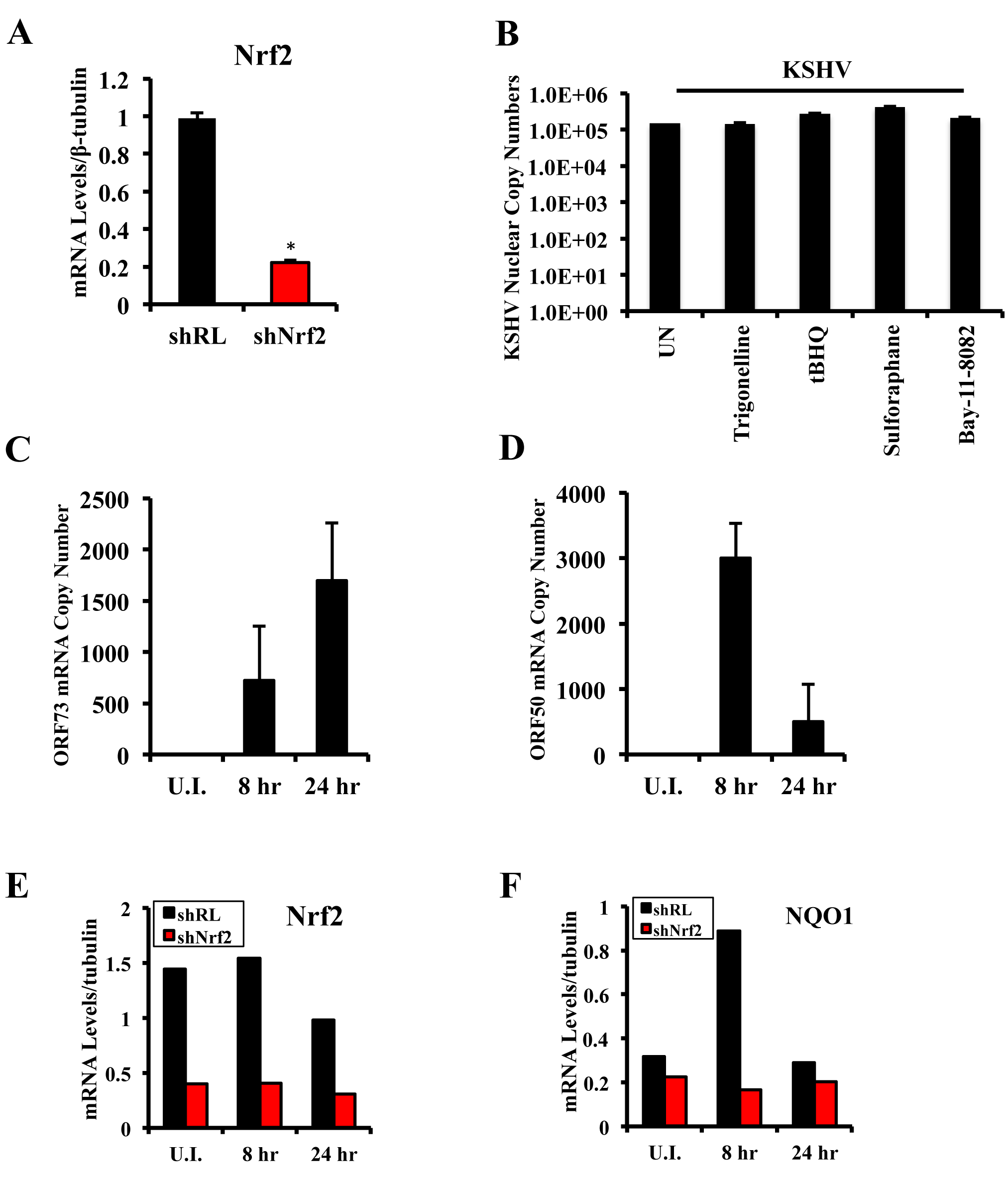

Supplement: Figure S8 — Nrf2 modulation and KSHV gene expression. A) Real-time PCR of Nrf2 mRNA to determine the efficiency of lentiviral knockdown for the nuclear delivery experiment. ShRL was arbitrarily set to 1 and the bars indicate mean ± SD for 3 replicates. * = p<0.05 when compared to shRL. B) Starved HMVEC-d cells were treated with the Nrf2 inhibitor Trigonelline (0.5 µM), Nrf2 inducers Sulforaphane (100 µM) and tBHQ (10 µM) and NF-κB inhibitor Bay-11-7082 (1 µM) for 4 hr prior to infection with KSHV (20 DNA copies/cell) for 2 hr. The nucleus-associated DNA was obtained using a DNeasy Blood & Tissue Kit (Qiagen) on nuclei isolated with Nuclei EZ Prep Nuclei Isolation Kit (Sigma-Aldrich) following the manufacturer's protocol. DNA real-time PCR was performed using ORF73 gene-specific primers to determine the levels of viral DNA. The absolute copy number was calculated from a standard curve obtained by real-time PCR of known standards with known concentrations of ORF73. Bars indicate mean ± SD for 3 independent replicates. C–D) Starved HMVEC-d cells were infected with KSHV (20 DNA copies/cell) prior to RNA isolation. One-step real-time PCR was performed on the viral genes C) ORF73 and D) ORF50. The absolute copy number was calculated from a standard curve obtained by real-time PCR of RNA standards of ORF73 or ORF50 with known concentrations. Bars indicate mean copy number ± SD of 3 independent replicates. E–F) Real-time PCR using specific primers for Nrf2 and NQO1 on RNA isolates of cells described in experiments under Figures 9E and F. Nrf2 PCR was performed to verify the knockdown in shNrf2 cells and NQO1 to determine the induction of Nrf2 in shRL cells. (TIF) [file ppat.1004460.s008.tif]

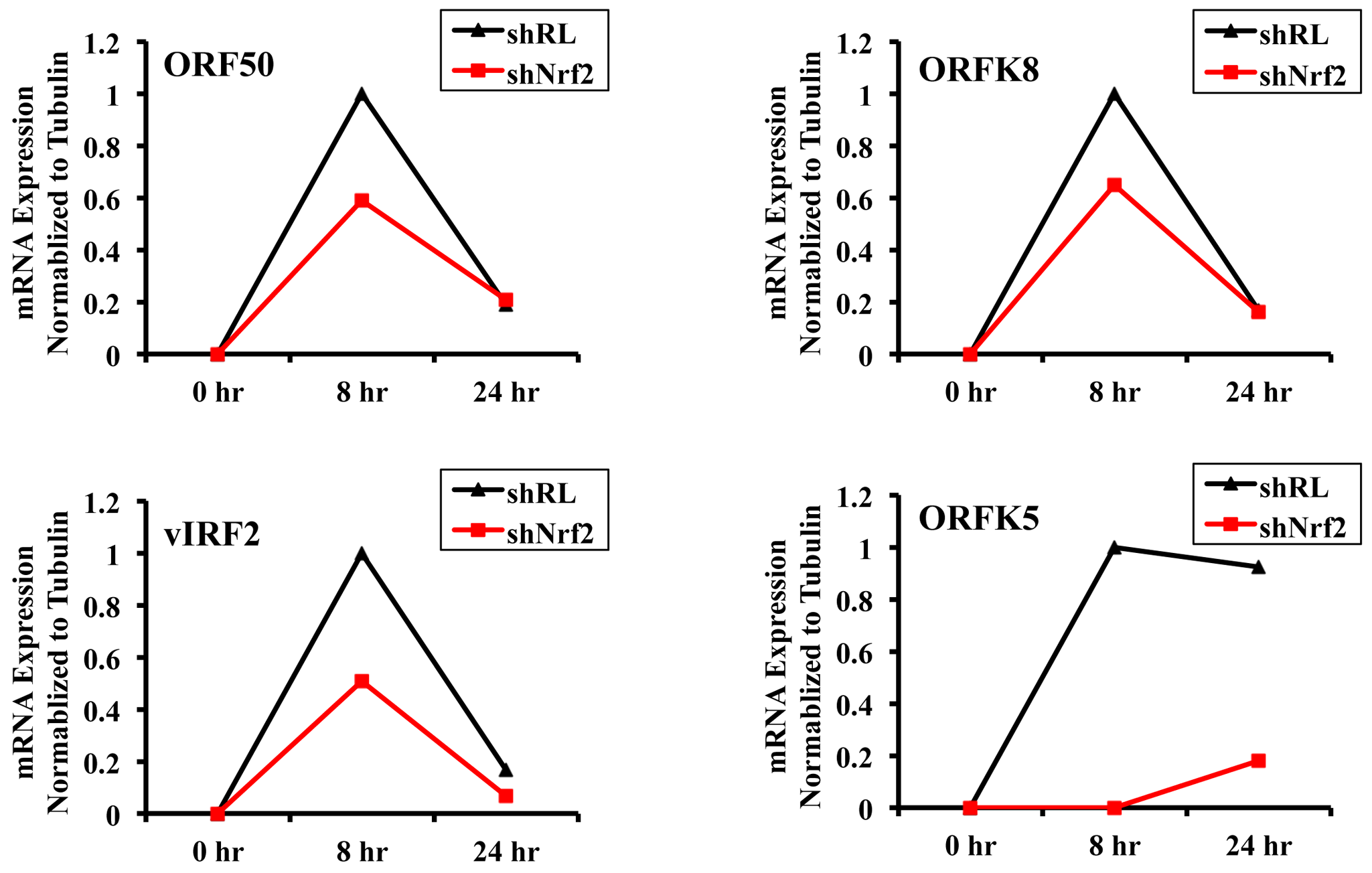

Supplement: Figure S9 — KSHV early lytic burst gene expression during Nrf2 knockdown. HMVEC-cells were initially transduced with shRL/shNrf2-containing lentiviral vectors for 72 hr prior to infection with KSHV (50 DNA copies/cell). The levels of various lytic genes such as ORF50, K8, K5 and vIRF2 were used using gene-specific primers by real-time RT-PCR. (TIF) [file ppat.1004460.s009.tif]

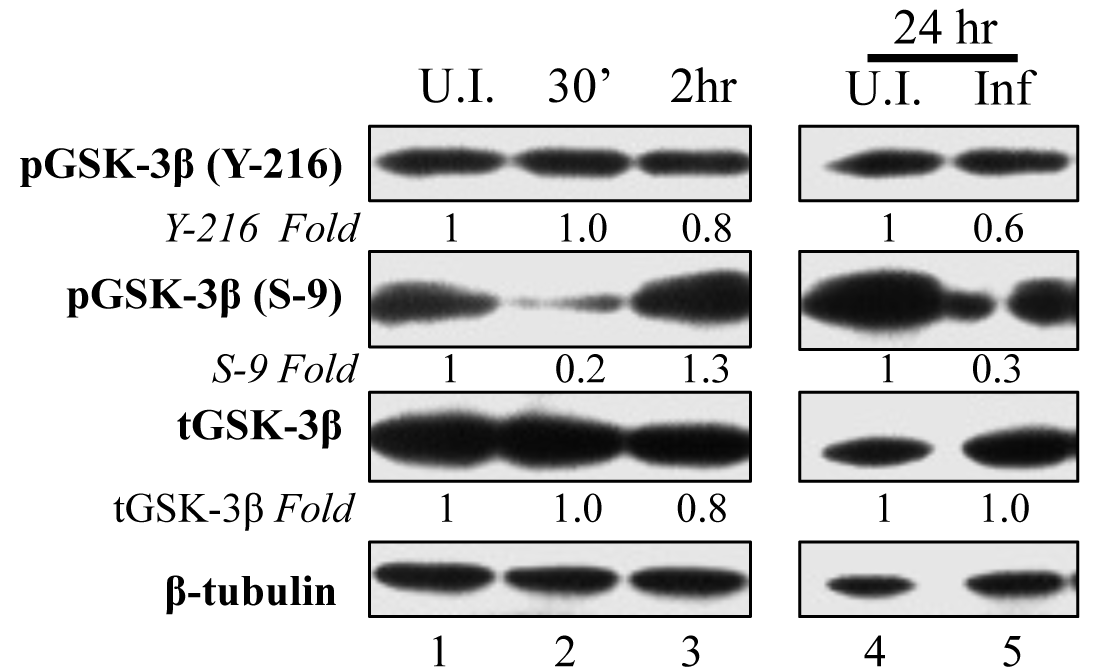

Supplement: Figure S10 — GSK-3β and its role in Nrf2 stability during de novo KSHV infection. HMVEC-d cells were infected with KSHV (20 DNA copies/cell) and immunoblotted for p62, pGSK-3β (Y-216), pGSK-3β (S-9) and tGSK-3β. Fold induction normalized to β-tubulin and relative to the uninfected (U.I.) condition (arbitrarily set to 1) are indicated. (TIF) [file ppat.1004460.s010.tif]
